# Unraveling the Toxicity of a Non-Microcystin-Producing Strain (CCIBt3106) of *Microcystis aeruginosa*: Ecotoxicological Effects on Aquatic Invertebrates

**DOI:** 10.3390/toxins17070321

**Published:** 2025-06-24

**Authors:** Éryka Costa Almeida, Fernanda Rios Jacinavicius, Rhuana Valdetário Médice, Rafaella Bizo Menezes, Larissa Souza Passos, Dominique Anderson, Jaewon Yoon, Elaine Dias Faria, Camila Manoel Crnkovic, Ana Lúcia Fonseca, Theodore Henry, Ernani Pinto

**Affiliations:** 1 Department of Clinical and Toxicological Analyses, School of Pharmaceutical Sciences, University of São Paulo, Sao Paulo 05508-000, Brazil; fjacinavicius@usp.br (F.R.J.); medicerv@usp.br (R.V.M.); larissapassos@usp.br (L.S.P.); 2 Laboratory of Cellular and Molecular Biology, Center for Nuclear Energy in Agriculture, University of São Paulo, Piracicaba 13400-970, Brazil; 3 Laboratory of Environmental Biogeochemistry, Center for Nuclear Energy in Agriculture, University of São Paulo, Piracicaba 13400-970, Brazil; rafabizo@usp.br; 4 Center for Marine Biodiversity & Biotechnology (CMBB), Institute of Life and Earth Sciences (ILES), School of Energy, Geoscience, Infrastructure and Society (EGIS), Heriot-Watt University, Edinburgh EH14 4AS, UK; dma3@hw.ac.uk (D.A.); t.henry@hw.ac.uk (T.H.); 5 Department of Biochemical and Pharmaceutical Technology, School of Pharmaceutical Sciences, University of São Paulo, Sao Paulo 05508-000, Brazil; jaewon@usp.br (J.Y.); camilavic@usp.br (C.M.C.); 6 Natural Resources Institute, Federal University of Itajubá, Itajuba 37500-903, Brazil; elainedfaria@gmail.com (E.D.F.); albadan.fonseca@gmail.com (A.L.F.); 7 Food Research Center (FoRC-CEPID), University of São Paulo, Sao Paulo 05508-080, Brazil; 8 Center for Carbon Research in Tropical Agriculture, University of São Paulo, Piracicaba 13418-900, Brazil

**Keywords:** cyanobacteria, secondary metabolites, ecotoxicology, *Daphnia similis*, *Parhyale hawaiensis*, non-microcystin-producing strain, LC-MS/MS, bioactive compounds

## Abstract

Cyanobacterial blooms are becoming increasingly frequent and intense worldwide, often dominated by *Microcystis aeruginosa*, a species capable of producing a wide array of bioactive metabolites beyond microcystins. This study evaluates the ecotoxicological potential of a non-microcystin-producing strain, *M. aeruginosa* CCIBt3106, using acute immobilization assays with three microcrustacean species: *Daphnia similis*, *Artemia salina*, and *Parhyale hawaiensis*. Biomass was extracted using solvents of varying polarity, and selected extracts (aqueous and 50% methanol) were further fractionated and analyzed via high-resolution liquid chromatography–tandem mass spectrometry (HR-LC-MS/MS). Significant toxicity was observed in *D. similis* and *P. hawaiensis*, with EC_50_ values ranging from 660 to 940 µg mL^−1^. Metabolomic profiling revealed the presence of chemically diverse metabolite classes, including peptides, polyketides, and fatty acyls, with putative annotations linked to known bioactivities. These findings demonstrate that cyanobacterial strains lacking microcystins can still produce complex metabolite mixtures capable of inducing species-specific toxic effects under environmentally relevant exposure levels. Overall, the results highlight the need to expand ecotoxicological assessments and monitoring frameworks to include non-microcystin cyanobacterial metabolites and strains in water quality management.

## 1. Introduction

Cyanobacteria are photosynthetic prokaryotes central to aquatic ecosystems, contributing to primary production and nutrient cycling. However, their capacity to form harmful algal blooms (cyanoHABs) has raised global concern due to negative effects on water quality, biodiversity, and public health [1,2]. These blooms release a wide variety of secondary metabolites, some of which are potent toxins such as microcystins—hepatotoxic cyclic peptides predominantly produced by *Microcystis aeruginosa* [3,4]. Consequently, much of the current research has focused on microcystin-producing strains, while strains that do not produce microcystins are often categorized as non-toxic, despite reports of significant bioactivity [3,5,6,7].

*M. aeruginosa* strain CCIBt3106 is a notable example of a microcystin-free strain. It has previously been shown to produce a variety of cyanopeptides such as aeruginosins and microginins, compounds with known bioactivities including protease inhibition and cytotoxicity [8]. Recent studies, such as those by Zhou et al. [7]., show that both microcystin-producing and microcystin-free strains of *M. aeruginosa* can pose a threat to aquatic ecosystems. This is because, although both strains share most metabolites, their relative concentrations and specific metabolic activities differ significantly, particularly during different growth phases. Moreover, microcystin-free strains of *M. aeruginosa* exhibit heightened physiological sensitivity to exogenous microcystins, which act not only as hepatotoxins but also as signaling molecules affecting metabolic regulation. These findings challenge the prevailing assumption that microcystin-free strains pose minimal ecological risk, emphasizing the importance of comprehensive toxicity assessments that go beyond microcystin quantification [9].

These findings underline the need to better characterize the full range of secondary metabolites beyond microcystins. The chemical diversity of cyanobacteria encompasses over 3000 known structures, including cyanopeptides, alkaloids, macrolides, and fatty acid derivatives [10,11,12]. These metabolites are not only structurally diverse but also biologically active, playing significant roles in ecological interactions and posing potential risks to aquatic organisms and human health. While microcystins remain among the most studied cyanobacterial toxins [13], recent findings reveal that microcystin-free strains of *M. aeruginosa* also release a wide range of bioactive compounds, some of which exhibit notable estrogenic activity [14]. Nonetheless, the metabolomic composition and toxicological significance of secondary metabolites from microcystin-free strains remain poorly understood, underscoring the importance of further studies to elucidate their environmental and human health implications.

To investigate the full spectrum of potential toxicity, cyanobacterial extracts are often obtained using solvents of varying polarity, ranging from polar solvents like water to more lipophilic ones, such as dichloromethane and methanol [8,15,16]. Sequential solvent extraction and polarity-based fractionation (e.g., with isopropyl alcohol gradients) can isolate compounds of different chemical classes and improve bioactivity assessments [17]. When coupled with metabolomic profiling, these approaches offer a powerful strategy for identifying novel or underexplored metabolites with ecotoxicological relevance.

The ecological impacts of cyanometabolites are particularly concerning for primary consumers such as microcrustaceans, which play critical roles in aquatic food webs. Model organisms, such as the freshwater cladoceran *Daphnia similis*, the marine crustacean *Artemia salina*, and the brackish- or marine-dwelling amphipod *Parhyale hawaiensis*, are widely used in ecotoxicology due to their sensitivity to pollutants and their representativeness of various aquatic environments [18,19,20]. Their responses to complex metabolite mixtures offer valuable insight into species-specific vulnerabilities and ecosystem-level risks.

This study investigates the acute toxicity (after 24 h exposure) of extracts and fractions from *M. aeruginosa* CCIBt3106 in these three model species, integrating ecotoxicological assays with untargeted high-resolution metabolomics (LC-MS/MS). Our objective is to characterize the bioactivity of non-microcystin metabolites and elucidate their potential ecotoxicological impacts. By highlighting the toxic potential of “non-toxic” cyanobacteria, this work challenges conventional paradigms and informs more comprehensive monitoring strategies in the context of increasing cyanoHAB events driven by eutrophication and climate change.

## 2. Results and Discussion

This study investigated the ecotoxicological potential of a non-microcystin-producing strain of *M. aeruginosa* (CCIBt3106) by combining toxicity assays with aquatic crustaceans from diverse ecological habitats and comprehensive metabolomic profiling. The approach combined polarity-targeted extraction, fractionation, acute toxicity testing, and high-resolution LC-MS/MS-based annotation to identify putative cyanobacterial metabolites associated with biological effects. The primary objective was to investigate alternative mechanisms of cyanobacterial toxicity and assess the ecological relevance of non-microcystin cyanometabolites in shaping aquatic food web dynamics.

### 2.1. Overview of Biomass and Extraction Yields

Details on biomass yield and extraction efficiency for *M. aeruginosa* strain CCIBt3106 are provided in Supplementary Tables S1 and S2. The high biomass yield (43.31 g dry weight over one year) and cell density (4.22 × 10^7^ cells mL^−1^) achieved during continuous cultivation of *M. aeruginosa* CCIBt3106 ensured not only the feasibility of repeated extraction and fractionation but also reflected bloom-like productivity levels under controlled conditions. These values are ecologically relevant, as they approximate the cell densities typically observed during intense natural blooms (up to 10^7^ cells mL^−1^) [21]. Such dense populations increase the likelihood of cyanometabolite accumulation in aquatic environments, representing a realistic model for evaluating bloom-associated toxicity. Additionally, the reproducibility of biomass production across multiple culture cycles reinforces the robustness of the toxicological and metabolomic analyses.

### 2.2. Extracts’ Toxicity Assessment in Aquatic Microcrustaceans

The acute toxicity of CCIBt3106 extracts was evaluated based on EC_50_ or LC_50_ values, defined as the concentration required to immobilize or kill 50% of the test organisms after 24 h (Table 1). *D. similis* was more sensitive to polar extracts, particularly the aqueous extract (EC_50_ = 740 µg mL^−1^) and 50% methanol extract (940 µg mL^−1^), values corresponding to environmentally relevant bloom densities (~7.4 × 10^7^ cells mL^−1^). *P. hawaiensis* showed higher sensitivity to methanol-based extracts, especially 100% methanol (LC_50_ = 600 µg mL^−1^), indicating a role for amphiphilic or lipophilic metabolites. In contrast, *A. salina* exhibited no measurable toxicity to any extract (EC_50_ > 1000 µg mL^−1^).

**Figure 1:**
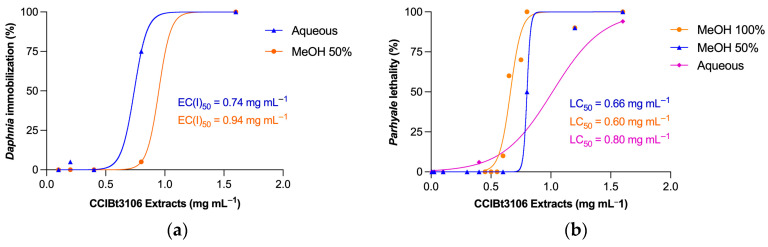
Dose-response curves for (**a**) *Daphnia similis* and (**b**) *Parhyale hawaiensis* exposed for 24 h to crude extracts from *Microcystis aeruginosa* CCIBt3106 (a non-microcystin-producing strain). EC_50_: effective concentration that immobilizes 50% of the test organisms; LC_50_: lethal concentration for 50% of the test organisms; MeOH: methanol.

These species-specific differences in sensitivity highlight physiological and ecological traits, such as differences in osmoregulation, membrane permeability, and feeding strategies. The dose–response curves for *D. similis* and *P. hawaiensis* (Figure 1) illustrate these patterns. Although *A. salina* was less responsive, its inclusion supports broader ecological comparisons across trophic levels.

The observed toxicity patterns are consistent with grazer–cyanobacteria interactions. Cyanobacteria are known to produce secondary metabolites that inhibit zooplankton grazing. Previous studies have shown that even microcystin-free strains can generate oligopeptides toxic to *Daphnia*, such as during *Planktothrix* blooms in European lakes [22,23]. The high sensitivity of *D. similis*, especially when compared to less susceptible species like *Moina micrura* (LC_50_ = 41,100 µg mL^−1^) [24], underscores the importance of species selection in ecotoxicological testing [25].

*P. hawaiensis* appears particularly suitable as a sentinel species for detecting lipophilic cyanobacterial metabolites, likely due to its benthic ecology and physiological traits favoring the uptake of less polar compounds. In contrast, the absence of measurable toxicity in *A. salina* may reflect either detoxification mechanisms or exposure below toxicity thresholds.

The lack of toxicity in the dichloromethane–methanol (1:1) extract (LC_50_ > 1000 µg mL^−1^) further supports a link between polarity and bioactivity. This trend is reinforced by the fractionation data (Section 2.3), where mid- and high-polarity fractions exhibited the greatest toxicity. These findings underscore the importance of solvent selection in chemical screening.

### 2.3. Fraction Toxicity and Chemical Polarity

**Figure 2:**
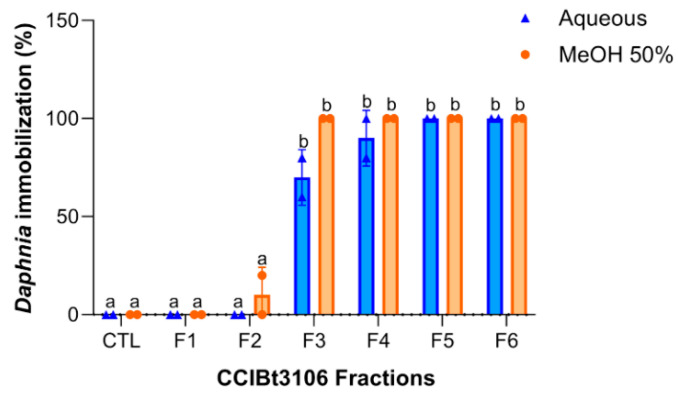
*Daphnia similis* immobilization (mean ± SD) after 24 h exposure to 150 μg mL^−1^ fractions of the aqueous (in blue) and MeOH 50% extracts (in orange) from *Microcystis aeruginosa* CCIBt3106 (a non-microcystin-producing strain). SD: standard deviation, CTL: control (only culture medium), F1: 0% IPA, F2: 20% IPA, F3: 40% IPA, F4: 70% IPA, F5: 90% IPA, and F6: 100% IPA. IPA: isopropyl alcohol; MeOH: methanol. Different letters (a, b) indicate statistically significant differences between treatments (ANOVA, *p* < 0.05).

Acute toxicity tests using *D. similis* revealed distinct patterns of immobilization across the fractions obtained from the aqueous and 50% methanol extracts of *M. aeruginosa* CCIBt3106 (Figure 2). Fractions F1 and F2, eluted with 0% and 20% isopropanol (IPA), respectively, showed no significant toxicity compared to the control (spring water only), with immobilization rates below 10%. In contrast, fractions F3 to F6, corresponding to intermediate- and high-polarity eluents (40–100% IPA), caused significantly higher immobilization rates, ranging from 70% to 100%, in both extracts.

This toxicity profile indicates that bioactive compounds were primarily recovered in moderately polar to highly polar fractions. The absence of significant differences between the aqueous and methanolic extract fractions suggests that solvent polarity had a greater influence on the separation of active compounds during fractionation than on their extraction from the biomass. This pattern aligns with earlier findings [26] and suggests that the most bioactive compounds in CCIBt3106 are best recovered using moderately polar solvents.

**Figure 3:**
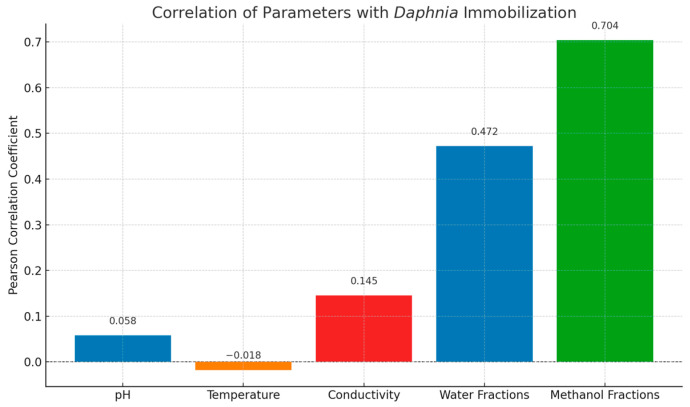
Pearson correlation coefficients between toxicity (*Daphnia* immobilization) and environmental/chemical parameters evaluated in the test.

To better understand the drivers of *D. similis* immobilization, a Pearson correlation analysis was performed between observed toxicity and a range of variables, including physicochemical parameters (pH, temperature, and conductivity) as well as the intensities of bioactive fractions (aqueous and methanolic). As shown in Figure 3, immobilization rates were strongly correlated with the methanolic (r = 0.704) and, to a lesser extent, aqueous (r = 0.472) fractions, while correlations with pH (r = 0.058), temperature (r = –0.018), and conductivity (r = 0.145) were weak or negligible.

These results indicate that the ecotoxicological responses were driven primarily by the chemical constituents of the extracts rather than basic environmental conditions. The stronger correlation observed with methanol fractions aligns with our LC-MS/MS findings, which revealed a high abundance of moderately polar to non-polar metabolites, such as fatty acyls, prenol lipids, and aromatic compounds, in these samples. Together, these data reinforce the hypothesis that bioactive secondary metabolites, rather than external abiotic factors, play a central role in mediating the observed immobilization effects on *D. similis*.

### 2.4. Metabolomic Analysis

LC-MS/MS-based metabolomics was employed to investigate the chemical diversity and potential drivers of toxicity in the extracts and fractions of *M. aeruginosa* CCIBt3106. The next subsections highlight the structural organization, class-level trends, and key compound annotations revealed through this approach.

#### 2.4.1. Molecular Networking and Class-Level Diversity

**Figure 4:**
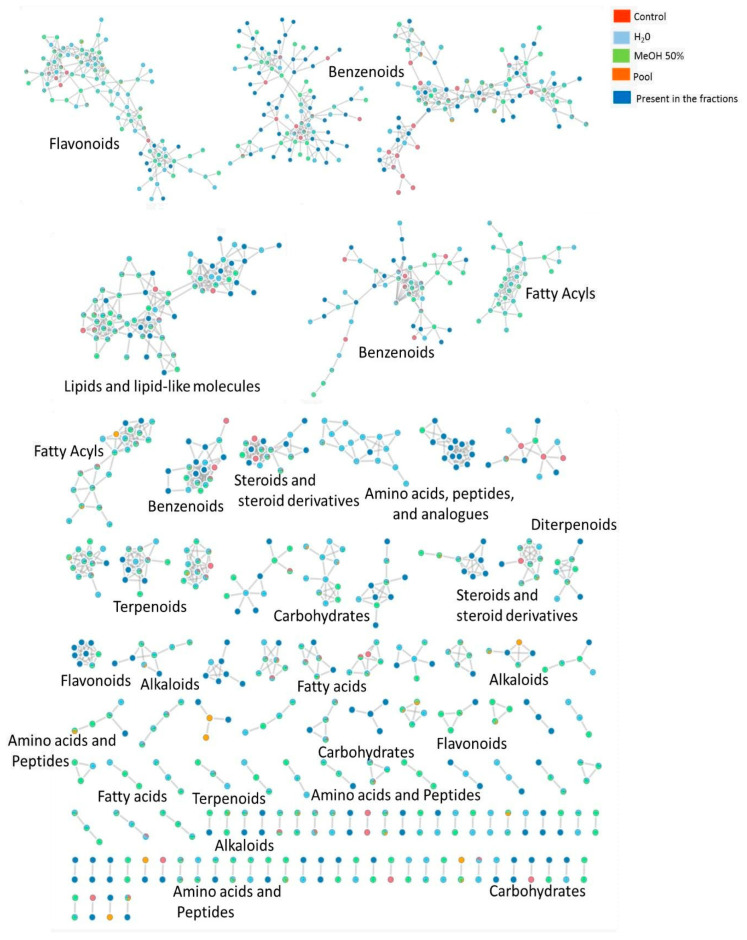
Molecular network generated from LC-MS/MS data of *Microcystis aeruginosa* CCIBt3106 extracts and fractions using GNPS and visualized in Cytoscape. Nodes represent precursor ions (*m*/*z*), and edges indicate spectral similarity. Node colors indicate the sample of origin: red for control (only spring water), light blue for H_2_O extract, green for MeOH 50% extract, pink for the pooled samples, and dark blue for features present in at least one aqueous or MeOH 50% fraction. Chemical classes were assigned to clusters based on GNPS libraries.

Classical molecular networking (GNPS) revealed structurally related compound families as distinct clusters across the extracts and fractions of *M. aeruginosa* CCIBt3106. As shown in Figure 4, annotated clusters encompassed chemical classes such as flavonoids, fatty acids, alkaloids, and terpenoids. Notably, key nodes were shared between aqueous and methanolic samples, reflecting the broad polarity range of certain metabolite families.

To further assess the influence of extraction solvents and distinguish extractable metabolites from background signals, Figure S1 presents a molecular network colored by sample type—including extracts obtained with H_2_O, MeOH 50%, MeOH 100%, and DCM:MeOH (1:1), as well as the control sample (spring water from the *D. similis* assay medium). The partially overlapping yet distinct distribution of clusters confirmed that solvent polarity plays a critical role in shaping the recovered metabolite profiles.

Feature distribution analysis (Figure S2) indicated that fractions F2 and F4 from the 50% methanol extract and F3 from the aqueous extract exhibited the highest chemical complexity. However, despite their richness, these were not necessarily the most toxic. Fractions classified as toxic (F3–F6), which induced high immobilization in *D. similis* (Figure 2), displayed slightly fewer features. This suggests that toxicity may be driven by specific bioactive metabolites rather than overall chemical abundance, reinforcing the need for compound-specific investigations.

#### 2.4.2. Toxicity-Driven Feature Distribution

To refine this observation, a targeted analysis of putatively annotated *m*/*z* features across chemical classes was performed. Table S3 summarizes representative *m*/*z* features grouped by chemical class and toxicity association. Metabolites uniquely detected in toxic fractions included flavonoids, fatty acyls, and prenol lipids, aligning with prior evidence of bioactivity in cyanobacteria. Conversely, phenol ethers and certain coumarins were restricted to non-toxic samples, indicating a likely lack of contribution to observed immobilization. Some ubiquitous classes, such as steroids and benzene derivatives, were present across both toxic and non-toxic samples, potentially reflecting their structural diversity or widespread metabolic roles. These findings support the hypothesis that only specific subclasses or individual analogues within broader chemical categories may drive toxicity.

**Figure 5:**
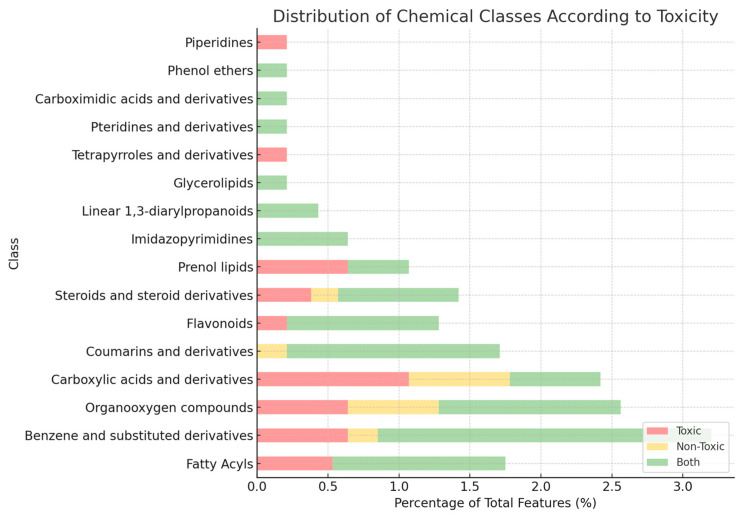
Relative distribution of chemical classes in *Microcystis aeruginosa* CCIBt3106 fractions using GNPS ClassyFire-based annotation. Features are grouped by their presence in toxic fractions (red), non-toxic fractions (yellow), or both (green), as determined from acute bioassays with *Daphnia* similis.

GNPS and ClassyFire-based classification (Figure 5) revealed the most frequently annotated chemical classes: fatty acyls (5.2%); benzene and substituted derivatives (4.4%); organooxygen compounds (3.3%); and amino acids, peptides, and analogues (3.0%). Despite these insights, over 70% of detected features remained unannotated or unclassified, underscoring the chemical novelty of this *Microcystis* strain and the limitations of current spectral libraries. These findings highlight the value of combining untargeted metabolomics with ecotoxicological bioassays to prioritize novel cyanobacterial metabolites for further investigation.

**Figure 6:**
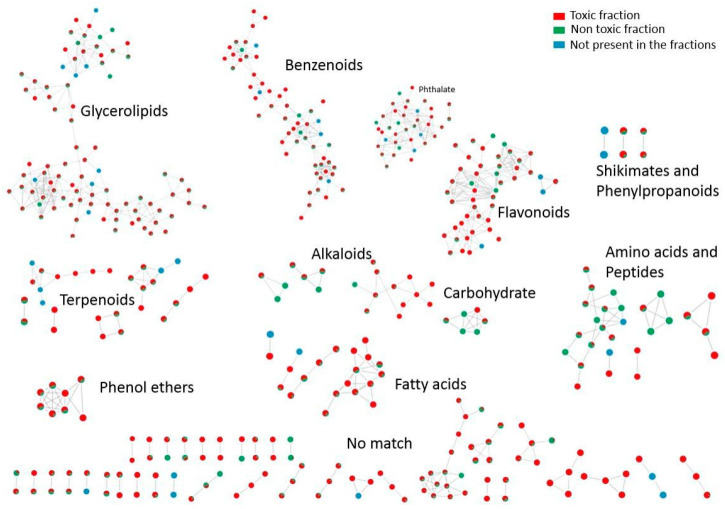
Molecular network showing the distribution of LC-MS/MS features across fractions classified as toxic or non-toxic based on bioassays with *Daphnia similis*. Nodes represent precursor ions (*m*/*z*), with red indicating presence in at least one toxic fraction, green in non-toxic fractions, and blue for features not detected in any fraction. Annotations of chemical classes were obtained through GNPS and include clusters of flavonoids, alkaloids, fatty acids, and terpenoids.

These toxic-specific clusters often occupied distinct regions of the molecular network (Figure 6), supporting a modular organization of bioactivity-related chemistry. Figure 6 illustrates the distribution of molecular features across fractions classified as toxic or non-toxic based on immobilization responses in *D. similis*. Clusters containing features found exclusively or predominantly in toxic fractions included candidates such as flavonoids and lipophilic compounds, suggesting that specific chemical groups may contribute to observed biological effects. In contrast, features restricted to non-toxic fractions tended to occupy distinct network regions, reinforcing a chemically driven separation between bioactive and inactive samples.

To consolidate the metabolomic and ecotoxicological findings, a multivariate statistical analysis was employed to explore how chemical composition and extraction polarity relate to observed biological effects. Redundancy analysis (RDA) and cloud plot visualization were used to evaluate the correlation between metabolite profiles and the toxicity outcomes observed in *D. similis*.

**Figure 7:**
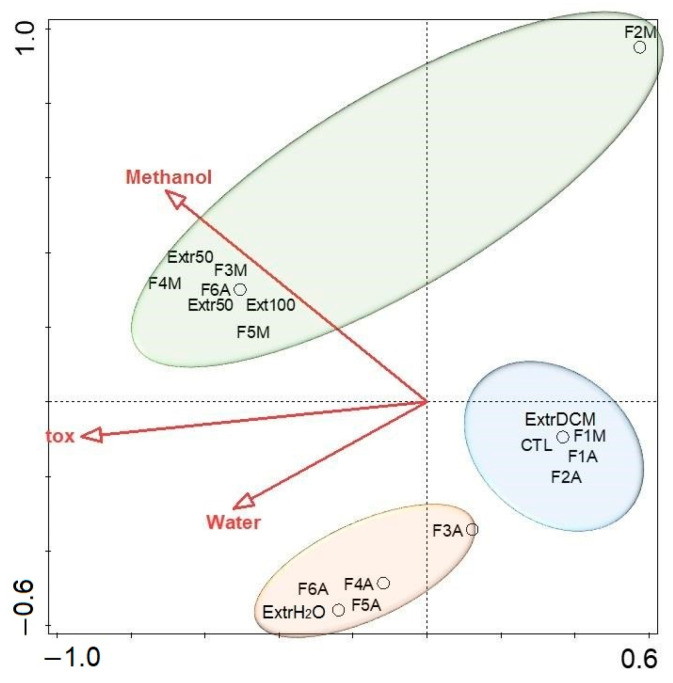
Redundancy analysis (RDA) biplot showing the relationship between toxicity, solvent polarity, and sample grouping. Toxicity vectors align with aqueous fractions, reinforcing chemical partitioning and biological activity. ExtrH_2_O = aqueous extract; Extr50 = 50% methanol extract; Ext100 = 100% methanol extract; ExtrDCM = dichloromethane–methanol 1:1 extract; F1-F6 = fractions; A = aqueous extract; M = 50% methanol extract; CTL = control solution (only spring water); tox = toxicity (*Daphnia similis* immobility).

**Figure 8:**
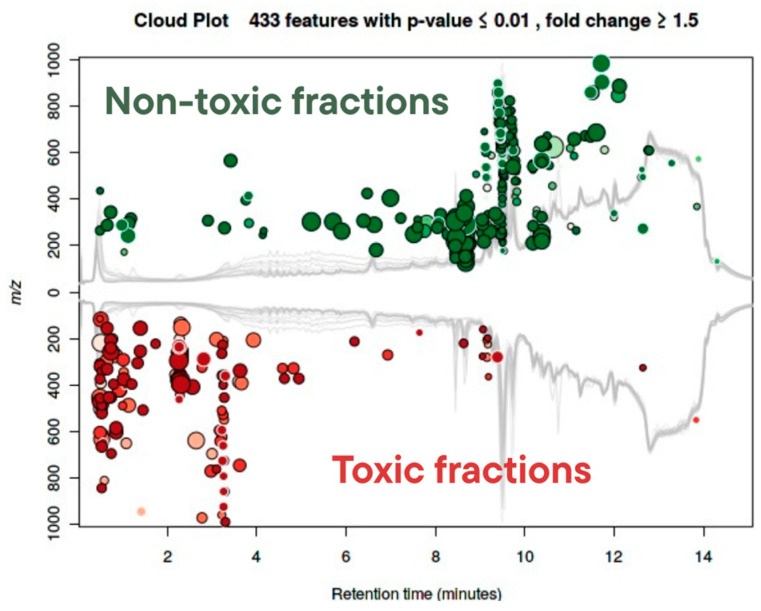
Cloud plot of differential metabolic features between toxic and non-toxic fractions. Each dot represents a metabolite with a significant difference (*p* ≤ 0.01; fold change ≥ 1.5). Red and green colors indicate enrichment in toxic and non-toxic fractions, respectively. Data are plotted by *m*/*z* versus retention time, with circle size proportional to effect size.

The RDA (Figure 7) revealed a clear segregation of samples: aqueous and 50% methanol fractions aligned closely with the toxicity vector, while low-polarity extracts (e.g., DCM: MeOH and F1 fractions) clustered away from toxic responses. This pattern reflects the role of moderately polar solvents in enriching compounds associated with biological activity and supports prior fractionation results (Figure 2) showing that toxicity was concentrated in intermediate to high-polarity eluents (F3–F6). Similarly, the cloud plot (Figure 8) identified 433 statistically significant features (*p* ≤ 0.01; fold change ≥ 1.5) differentially abundant between toxic and non-toxic fractions. These enriched features spanned a broad *m*/*z* range and clustered predominantly between 2 and 10 min retention time, highlighting discrete chemical signatures associated with toxicity rather than diffuse chemical complexity. While Table S3 provides a class-level snapshot of toxicity associations, the cloud plot quantitatively validates and visualizes the scale and distribution of bioactivity-linked metabolic differences. Together, these findings support a model where toxicity arises from defined chemical signatures, not from overall metabolite abundance.

These analyses underscore that bioactivity in *M. aeruginosa* CCIBt3106 is not driven by generalized metabolite richness but by specific, chemically defined groups enriched under certain extraction conditions. This reinforces the value of polarity-targeted metabolomics for identifying ecologically relevant compounds. These results lay the groundwork for evaluating the broader ecological and environmental relevance of the observed metabolite–toxicity relationships. These chemically resolved toxicity patterns raise important ecological questions, particularly regarding how cyanobacterial metabolite profiles influence environmental risk.

#### 2.4.3. Putative Metabolite Annotations and Diagnostic Fragmentation Patterns

Three compounds were putatively annotated in the extracts and fractions of *M. aeruginosa* CCIBt3106 based on LC-MS/MS data (Table 2). Following the framework by Schymanski et al. [27], confidence levels were assigned to a nucleoside (inosine), a pterin derivative (microcystbiopterin), and a novel microginin-like cyanopeptide, each representing distinct structural classes relevant to cyanobacterial metabolism.

Inosine, a purine nucleoside involved in stress signaling and purinergic modulation [28], was detected in all extracts and fractions, with a mean *m*/*z* of 269.0889 [M+H]+ and a retention time of 1.48 min. The predicted molecular formula (C_10_H_12_N_4_O_5_) matched an entry in CyanoMetDB with a low mass error (1.15 ppm). MS^2^ spectra showed strong similarity to library references, including diagnostic fragments (*m*/*z* 82.04, 94.04, 110.04, 119.04, 137.05; Figure S3), supporting a Level 2a annotation. Inosine was particularly abundant in F2 H_2_O, a non-toxic fraction, though it also appeared in toxic ones (e.g., F3 H_2_O and F5 MeOH 50%). Its widespread distribution and lack of correlation with toxicity suggest it may act as a background metabolite or reflect co-extraction with toxic compounds rather than direct bioactivity.

Another compound, *m*/*z* 414.1622 [M+H]+ (RT 3.1 min), was putatively annotated as microcystbiopterin B or E—a pterin-like compound implicated in photoprotection [29]. The formula C_16_H_23_N_5_O_8_ was predicted by SIRIUS and matched a CyanoMetDB entry (mass error −0.70 ppm). Although reference MS^2^ spectra were unavailable, the experimental fragmentation pattern (*m*/*z* 124.05, 147.07, 178.07, 192.09, 220.08, and 238.09; Figure S6) supported a Level 2b annotation. This feature appeared in all major extracts (MeOH 50%, MeOH 100%, and DCM: MeOH) and fractions F1–F4. While its presence in toxic samples suggests possible involvement in bioactivity, it is unlikely to act as a sole driver of the observed toxicity, though synergistic roles remain plausible.

A third feature (*m*/*z* 591.3587 [M + H]^+^; RT 5.02 min) was proposed as a novel microginin analogue. The MS^2^ spectrum (Figure S8) contained canonical fragments (*m*/*z* 128.9621, 345.9130, 333.3609, 337.0260, and 358.9687), consistent with known microginins [30,31]. The predicted formula, C_31_H_50_N_4_O_5_S, showed high accuracy (1.15 ppm). Although no exact spectral match was found, diagnostic ions strongly supported its classification as a microginin-like compound (Level 2b). Notably, this feature was most abundant in the toxic MeOH 50% and 100% extracts, while present at lower levels in aqueous fractions.

Microginins are cyanobacterial peptides with potent inhibitory effects on proteolytic enzymes such as ACE, trypsin, and aminopeptidases [32]. Their known biological activity and abundance in toxic samples point to a plausible contribution to observed immobilization. Figures S4 and S9 illustrate their concentration patterns across extracts and fractions, reinforcing their possible role in toxicity either independently or through synergistic effects.

The mass accuracy of all annotated features (error range: −0.70 to +1.15 ppm) supports the reliability of identifications. Annotation levels (2a–2b) reflect high-confidence assignments either via library spectral matches or robust fragmentation patterns in the absence of references.

The abundance of these compounds partially mirrored toxicity profiles. The putative microginin (*m*/*z* 591.359) was consistently elevated in toxic samples, whereas inosine showed stable, low abundance and no apparent toxicity correlation. Microcystbiopterin (*m*/*z* 414.162) presented intermediate behavior, with higher levels in toxic extracts but also widespread presence, suggesting possible synergistic or indirect contributions.

**Figure 9:**
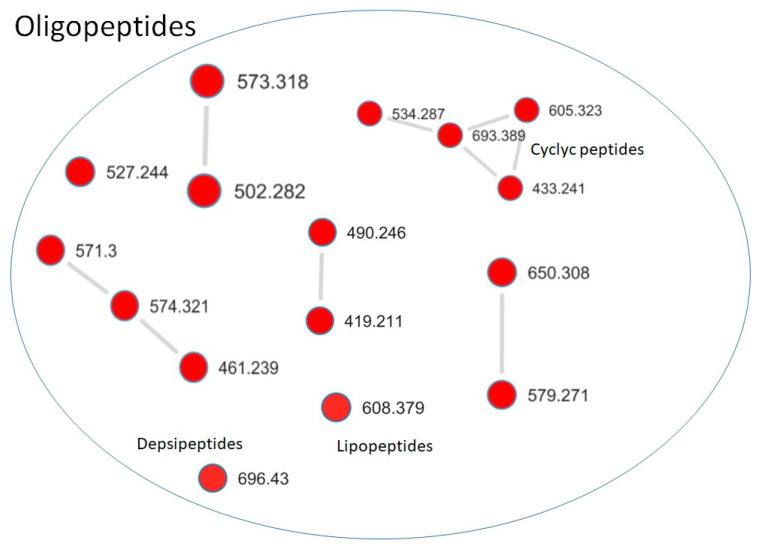
Molecular network highlighting a cluster of oligopeptides exclusively detected in toxic fractions of *Microcystis aeruginosa* CCIBt3106. Each node represents a precursor ion (*m*/*z*), and edges indicate spectral similarity. Red nodes correspond to features found only in toxic fractions, while the blue node represents a feature not detected in any fraction. Subclusters were manually annotated based on MS/MS fragmentation and network topology as likely corresponding to cyclic peptides, depsipeptides, and lipopeptides.

In addition to the annotated microginin-like compound, molecular networking revealed a distinct cluster of oligopeptides found exclusively in the toxic fractions (Figure 9). MS/MS spectra of these compounds (Table S4, Figures S10 and S11) showed fragment ions consistent with amino acid substructures, including *m*/*z* 70.0657 (Pro), 86.0968 (Leu/Ile), 102.0553 (Glu), 120.0809 (Phe), 129.0426 (Glu or derivatives), 136.0755 (Tyr), 140.0663 (Tyr-related), 231.0977 (Me-Tyr or homophenylalanine), 84.0814 (Lys), 115.0865 (Lys-related), and 201.0985 (Arg fragment), all common in cyanopeptides.

Fragments at *m*/*z* 197.0919 and 169.0967 match Thr-Ahp structures, typical of cyanopeptolins. The ion at *m*/*z* 100.1122 (Ahoa) supports the presence of microginin-like peptides. Several observed fragments correspond to known cyanopeptide markers described in the literature [30,33]: *m*/*z* 201.0985 (Arg-CO, anabaenopeptins), *m*/*z* 159.0911 (Trp, microviridins), *m*/*z* 181.1331 and 215.1167 (Ahp-containing cyanopeptolins), and *m*/*z* 86.0059 and 84.0448 (TzlN and MeOx, cyanobactins).

These fragmentation patterns are consistent with structural motifs commonly found in bioactive cyanobacterial peptides. Their restriction to toxic fractions and similarity to known cyanopeptides indicate a likely role in the observed ecotoxicity, either individually or through synergistic effects.

Although aeruginosins have been previously reported in strain CCIBt3106, they were not detected under the cultivation and extraction conditions employed in this study. While the same medium and temperature were used as in earlier reports, the cultures in our study were subjected to continuous aeration and harvested at different time points—factors that may have influenced the expression of secondary metabolites. In particular, the biosynthesis of aeruginosins involves oxygen-dependent tailoring enzymes such as AerH dioxygenase and is therefore susceptible to redox fluctuations. Oxygenation can alter cellular redox balance and photosynthetic efficiency, indirectly modulating metabolite production. Previous studies have shown that cyanobacteria maintain a chemically reduced microenvironment via sulfhydryl-rich mucilage, which buffers oxidative stress and supports specific biosynthetic pathways [34]. The disruption of this microenvironment by forced aeration may have suppressed the production of redox-sensitive compounds such as aeruginosins [35,36].

Collectively, these annotations highlight the complementarity of spectral analysis, in silico prediction, and bioactivity-guided interpretation in metabolomic studies. The diversity of detected chemical classes—ranging from nucleosides to cyanopeptides—reinforces the complex and dynamic metabolome of *M. aeruginosa* CCIBt3106. These metabolites emerge as promising candidates for future functional and toxicological validation. Their consistent association with bioactive fractions provides a foundation for exploring their ecological significance and potential risks to aquatic ecosystems.

#### 2.4.4. Environmental Implications and Chemical Risk

These results challenge the traditional focus on microcystins as the sole indicators of bloom toxicity. Although the studied strain (CCIBt3106) does not produce microcystins, it induced potent immobilization effects in *D. similis* and *P. hawaiensis*, particularly through moderately polar fractions such as those recovered with MeOH 50%. The detection of putative microginins, microcystbiopterins, and other cyanopeptides in these fractions provides mechanistic support for these effects, aligning with recent findings that microcystin-free *M. aeruginosa* strains can exert significant biological impacts through alternative metabolite pathways [5,6,7,9,14]. In particular, Cai et al. [9]. demonstrated that microcystin-free strains retain physiological sensitivity to exogenous microcystins, which may act as signaling molecules, further challenging the conventional toxic vs. non-toxic classification used in bloom assessments.

From an ecological standpoint, the implications are substantial. Crustaceans such as *Daphnia* and *Parhyale* are central to trophic transfer and top-down regulation in aquatic food webs. Their impairment could disrupt grazing pressure, nutrient cycling, and phytoplankton succession. The presence of protease inhibitors like microginins—known to interfere with digestive physiology in metazoans [32]—raises concerns about sublethal and chronic effects on zooplankton fitness, with cascading consequences for ecosystem stability.

Moreover, the enrichment of toxic features in moderately polar fractions reflects the physicochemical nature of bioactive metabolites. While solvent polarity itself is an analytical parameter, the ability of polar solvents to extract toxic compounds mirrors the environmental reality that such metabolites, once produced, can be released into aquatic systems. External factors such as temperature, light intensity, and UV radiation are known to modulate cyanobacterial metabolite production. Indeed, previous studies have shown that UV exposure can shift CCIBt3106’s metabolome toward nodularins, spumigins, and anabaenopeptins—bioactive compounds absent under standard growth conditions [8,29].

These metabolite shifts underscore the plasticity of cyanobacterial secondary metabolism in response to environmental stressors. The detection of microcystbiopterins—pterin-like compounds potentially involved in photoprotection [29]—in toxic fractions supports the idea that these metabolites may serve multifunctional ecological roles, including stress response, chemical defense, or grazer deterrence. Additionally, chemical classes enriched in toxic samples, such as flavonoids and fatty acyls, have been linked to cytotoxicity and membrane disruption in eukaryotic cells [37,38], indicating possible mechanistic pathways for the immobilization effects observed in crustaceans.

The EC_50_ values obtained for *D. similis* (e.g., 740 µg mL^−1^ for aqueous extracts) correspond to cyanobacterial cell densities commonly observed during natural blooms (~10^7^ cells mL^−1^) [21], suggesting that even microcystin-free strains could pose ecological risks at environmentally relevant concentrations. Such exposure could surpass WHO safety thresholds for microcystin-LR (1 µg L^−1^) despite the absence of that specific toxin [39]. These findings are consistent with broader reports linking non-microcystin metabolites to estrogenic, cytotoxic, and neuroactive effects in freshwater ecosystems [11,14].

The integration of untargeted metabolomics with ecotoxicological assays in this study advances the current understanding of chemical risks associated with cyanobacterial blooms. It highlights the need to expand regulatory frameworks beyond traditional toxin-centric models, incorporating bioactivity-based assessments and considering lesser-known cyanometabolites. As climate change and eutrophication intensify the frequency and duration of cyanoHABs, predictive and diagnostic tools grounded in chemical and functional diversity will be essential for effective freshwater monitoring, risk mitigation, and ecosystem protection.

## 3. Conclusions

This study provides robust evidence that cyanobacterial blooms dominated by *M. aeruginosa* strains non-microcystin-producing, such as CCIBt3106, can still exert significant ecotoxicological effects at environmentally relevant concentrations. Acute immobilization responses observed in *D. similis* and *P. hawaiensis*, particularly in response to aqueous and moderately polar methanol-based extracts and fractions, underscore the ecological risk posed by non-microcystin cyanometabolites—bioactive compounds that often go undetected in conventional monitoring programs.

The heightened sensitivity of *P. hawaiensis* to more lipophilic extracts highlights the importance of considering species-specific physiological traits and differential compound uptake in ecotoxicological testing. These findings also suggest that estuarine and euryhaline invertebrates may serve as useful bioindicators for detecting lipophilic cyanobacterial metabolites in transitional water systems.

Untargeted LC-MS/MS-based metabolomics revealed a chemically diverse set of metabolite classes enriched in toxic fractions, including peptides, fatty acyls, flavonoids, and pterin derivatives. Of particular interest were clusters of oligopeptides with fragmentation patterns characteristic of cyanopeptide families such as microginins, cyanopeptolins, anabaenopeptins, and microviridins. These classes are widely recognized for their inhibitory, cytotoxic, or membrane-disrupting properties and are known to interfere with key physiological processes in aquatic organisms. Their exclusive detection in toxic fractions, combined with diagnostic fragmentation consistent with modified amino acid residues, strongly supports their involvement in the observed biological effects, likely through additive or synergistic mechanisms.

The absence of microcystins in all extracts, despite measurable toxicity, reinforces the need to reassess bloom toxicity classification schemes. Current monitoring frameworks, which emphasize only canonical cyanotoxins, may significantly underestimate ecological risk, especially in blooms composed of metabolically diverse but non-microcystin-producing strains.

In the context of increasing eutrophication and climate-driven bloom intensification, these findings call for broader and more functionally informed surveillance strategies. Future efforts should focus on chronic toxicity testing, structure elucidation of key metabolite classes, and predictive ecological modeling to understand how these compounds propagate across trophic levels.

## 4. Materials and Methods

### 4.1. Microcystis aeruginosa (CCIBt 3106 Strain) Cultivation

The CCIBt 3106 strain of non-microcystin-producing *M. aeruginosa* was acquired from the Algae, Cyanobacteria, and Fungi Culture Collection of the Botany Institute (CCIBt), located in São Paulo, SP, Brazil. Originally isolated from the Salto Grande Reservoir in Americana-SP, Brazil (22°44′ S, 47°19′ W), this strain was cultivated in ASM-1 medium [40], under controlled conditions: pH 8.0 ± 0.3, temperature of 22.0 ± 0.5 °C, with a 12 h light/12 h dark photoperiod, and an irradiance of 22 ± 5 μmol photons m^−2^ s^−1^. The cultivation was conducted in cycles of approximately 40 days. At the end of each cycle, 10% of the total volume of the culture was used as inoculum for new flasks, ensuring the continuity of the process. The remaining culture was centrifuged, and the resulting biomass was stored at −20 °C for subsequent lyophilization and use in ecotoxicological tests.

### 4.2. Cell Counting

After approximately 40 days of growth, three 1 mL samples were aseptically extracted from the CCIBt 3106 cultures. These samples were then treated with 0.5 mL of Lugol solution for fixation. Cell counting was conducted in triplicate using a Fuchs–Rosenthal Chamber in a Zeiss Axiovert 135M optical microscope (Carl Zeiss, Göttingen, Germany). Cell density determination was based on the area encompassing 16 squares within the chamber. The number of cells within the counted area was multiplied by a factor of 5 × 10^3^ to express the cell density in cells mL^−1^ [41].

### 4.3. Extraction of Cyanobacteria Secondary Metabolites

The dry biomass of strain CCIBt3106 was weighed in Erlenmeyer flasks. A mass/volume ratio of 1:10 was used, with different solvents (ultrapure water, 50% methanol, 100% methanol, or dichloromethane–methanol 1:1 *v*/*v*) added to extract various classes of metabolites [42]. After an overnight extraction period (~15 h), the extracts were filtered into pre-weighed glass vials. The solvents were then dried using a nitrogen flow (for methanolic extracts) or subjected to lyophilization (for aqueous extracts). The flasks were weighed again to determine the yield of the extracts, and they were stored at −20 °C until the ecotoxicological tests.

### 4.4. Fractioning of the Toxic Extracts

For the dry extracts that exhibited toxicity toward *D. similis*, fractionation was performed using Diaion^®^ HP-20SS resin with a stepwise isopropanol–water gradient, following protocols established in cyanobacterial natural products [43,44]. The column was preconditioned with 10 mL of IPA, followed by 10 mL of ultrapure water. The compounds were eluted using a gradient of IPA by passing 20 mL of each IPA: H_2_O solvent mixture (0:100, 20:80, 40:60, 70:30, 90:10, and 100:0 IPA). Subsequently, the column was washed with 20 mL of ethyl acetate and 20 mL of acetone. Each fraction obtained from the solvent mixtures (fraction 1 to fraction 6, F1–F6) was collected, dried using a nitrogen flow, and stored at −20 °C until the ecotoxicological tests.

### 4.5. Acute Toxicity Test with Daphnia similis (Crustacea: Cladocera)

The acute toxicity tests with *D. similis* followed the guidelines from the Brazilian Association of Technical Standards, NBR 12713/2016 [45]. The dry extracts of CCIBt3106 (aqueous, 50% methanol, 100% methanol, and dichloromethane–methanol 1:1) were diluted in natural spring water. The organisms were exposed to CCIBt3106 extracts in concentrations of 0.100, 0.200, 0.400, 0.800, and 1.600 mg mL^−1^ for the aqueous, 50% methanol, and 100% methanol extracts, and 0.001, 0.010, 0.100, and 1.000 mg mL^−1^ for the dichloromethane–methanol 1:1 extract, along with a control group (natural spring water only). Then, twenty neonates aged between 6 and 24 h were distributed into four replicates, with five organisms in each test container containing 10 mL of the test solution. The fractions obtained from the toxic extracts were tested at a concentration of 0.150 mg mL^−1^. The organisms were incubated in a biological oxygen demand chamber (BOD) at a temperature of 22 °C, a photoperiod of 12 h light and 12 h dark, and without food administration. Immobilized or deceased organisms were quantified after 24 h of exposure. The test was considered valid when the percentage of immobilized or deceased organisms in the control group did not exceed 10% at the end of the experiment.

### 4.6. Acute Toxicity Test with Parhyale hawaiensis (Crustacea: Amphipoda)

Wild-type *P. hawaiensis* from *L’Institut de Génomique Fonctionnelle* in Lyon, France, were cultured in a circular flow system with artificial seawater (ASW) (38‰) consisting of 6–8 tanks (5 L each) and a 30 L sump, under abiotic conditions described by Artal et al. [46]. Partial water changes were performed every 2 weeks, and amphipods were fed twice weekly with a 1:1:1 mix of freeze-dried tubifex worms, fish flakes, and wheat germ pellets. Adult organisms were separated and placed in a 2.5 L plastic container filled with natural rocks (10–15 cm) for 72 h for acclimation.

Following the acclimation period, acute toxicity tests (LC_50_) were conducted. Ten replicates per concentration were prepared, placing one adult organism per well (10 mL of test solution) in 6-well plates. After range-finding tests, the organisms were exposed to CCIBt3106 extracts in concentrations of 0.00625, 0.025, 0.100, 0.300, 0.400, 0.600, 0.800, 1.200, and 1.600 mg mL^−1^ for the aqueous extracts; 0.450, 0.500, 0.550, 0.600, 0.650, and 0.750, 0.800, 1.200, and 1.600 mg mL^−1^ for 50% methanol; and 0.00625, 0.025, 0.100, 0.400, and 1.600 mg mL^−1^ for the extracts 100% methanol extract and dichloromethane–methanol 1:1, along with a control group (ASW only). The tests were maintained under the same conditions as the cultures. After 24 h of exposure, the mortality rate was checked for each concentration and tested extract.

### 4.7. Cytotoxicity Test with Artemia salina (Crustacea: Anostraca)

The procedures for culture, harvesting, and toxicity tests with *A. salina* were performed according to Metcalf et al. [2]. *A. salina* eggs were incubated in ASW and the system was artificially illuminated, aerated, and maintained at a constant room temperature of 25 °C for 24 h.

The dried CCIBt3106 extracts were diluted in 10.00 mg mL^−1^ dimethyl sulfoxide (DMSO) at concentrations of 0.01, 0.1, 1, 5, 10, 50, and 100 μg mL^−1^. To each well of the 96-well plate, 99 μL of ASW containing 10–20 *A. salina* nauplii and 1 μL of sample or control solution were added. The final concentrations were 0.0001, 0.001, 0.010, 0.050, 0.100, 0.500, and 1.000 mg mL^−1^, along with a negative (1% DMSO) and positive control (K_2_Cr_2_O_7_ 200 μg/mL). The entire experimental procedure was performed in triplicate to ensure the reliability of the results. After 24 h of exposure at 25 °C, the dead organisms were counted.

### 4.8. Toxicity Statistical Analysis

The dose–response data, expressed as percentages of mortality or immobility per replicate at each tested concentration and extract, were analyzed to evaluate the toxicological effects. Initially, the Kolmogorov–Smirnov (KS) test was applied to assess the normality of the data. Subsequently, the data were fitted to a dose–response curve using a non-linear regression model (*Logit*(*P*) *= μ + b*) appropriate for sigmoidal relationships typically observed in toxicity studies, where *logit*(*P*) expresses the log-odds of mortality, *μ* the intercept, and *b* the concentration coefficient. This analysis provided key toxicological parameters, including the predicted median lethal concentration (LC_50_) or the median effective concentration (EC_50_), calculated using the *logit*(*P*) with 95% confidence intervals to quantify toxicity levels (*P = e ^logit^*^(*P*)^*/*(1 *+ e ^logit^*^(*P*)^)). The dose–response curves were generated for each extract and organism to visualize the trends in toxicity.

### 4.9. LC-MS and Metabolomics Analysis

The analysis was conducted using a high-performance liquid chromatography system (Shimadzu© Prominence Liquid Chromatography, Kyoto, Japan) coupled with a high-resolution tandem mass spectrometer (MicroTOF-QII; Bruker Daltonics©, Billerica, MA, USA) equipped with electrospray ionization (ESI), configured as HPLC-ESI-QTOF-MS/MS. The method was adapted from Jacinavicius et al. [8] and Sanz et al. [47].

The tested samples, consisting of fractions and extracts from the CCIBt3106 strain, were the same as those prepared for the *D. similis* toxicity tests. These samples were diluted in culture water, which is essentially spring water. Additionally, control samples containing only the culture water were analyzed under the same conditions to account for potential components from the water itself. All samples were filtered through a 0.22 μm PVDF syringe filter and directly injected (10 µL) into the system.

Chromatographic separation was performed using a Luna Kinetex reversed-phase C18 column (100 Å, 50 × 2.1 mm, 2.6 µm particle size). The mobile phases consisted of Milli-Q water with 0.2% formic acid (phase A) and acetonitrile with 0.2% formic acid (phase B). A gradient elution program was applied, starting with 5% phase B and increasing linearly to 100% B from 0.01 to 12 min, maintaining 100% B from 12 to 13 min, and then returning to 5% B at 13.01 min and holding until the end of the run at 15 min. The flow rate was set at 0.6 mL min^−1^.

The ionization source was operated at a capillary potential of 3500 V, with nitrogen as the drying gas at 200 °C, a flow rate of 9.0 mL min^−1^, and a nebulizer pressure of 45.0 psi. The mass spectrometer was operated in auto MS/MS scan mode, with mass spectra acquired in both positive and negative ionization modes across a mass range of 50–1800 *m*/*z*.

### 4.10. Metabolite Identification

#### 4.10.1. Processing of Raw Data

The raw data generated by the mass spectrometer (.d format) were initially processed using Bruker Compass DataAnalysis^©^ 4.4 software (Bruker Daltonics, Bremen, Germany). This involved calibration and subsequent conversion of the files into the .mzXML format for compatibility with downstream analysis platforms. The processed data were then uploaded to the Global Natural Products Social Molecular Networking (GNPS) platform for further analysis [48].

#### 4.10.2. Molecular Networking

A classical molecular networking workflow was performed within the GNPS platform. The process involved filtering MS^2^ fragment ions to include only those within a range of ±17 Da from the precursor *m*/*z*. The six most intense fragment ions within a window of ±50 Da were selected for analysis. Precursor and fragment ion mass tolerances were both set to 0.02 Da. Molecular networks were constructed using a minimum cosine similarity score of 0.7 and at least six matched peaks. The resulting networks were visualized using Cytoscape^©^ 3.7.1 software [49]. Spectral library matches available in GNPS were employed for automatic metabolite annotation by comparing reference spectra with filtered data.

#### 4.10.3. Metabolite Annotation

Metabolite annotation combined accurate mass measurements, isotopic patterns, and MS/MS spectral similarity to putatively identify compounds. These were cross-referenced against databases like CyanoMetDB [50] and the scientific literature. Sirius 4 software was used to assist in compound annotation through in silico predictions [51].

Annotations were classified according to the confidence framework proposed by Schymanski et al. [27]. Level 1 represents confirmed structures, where matches with MS and MS2 spectra were validated using authentic reference standards and corroborated with orthogonal methods like nuclear magnetic resonance (NMR) when available. Level 2 annotations, indicating probable structures, were assigned based on either spectral library matches (2a) or diagnostic evidence (2b) when reference standards were unavailable. Level 3, assigned to tentative candidates, included suggested structures, such as isomers, when data were insufficient for exact identification. Level 4 was used for unequivocal molecular formulas confidently determined without structural information. Finally, Level 5 indicated compounds for which only the exact mass was determined, without enough data to propose a molecular formula.

### 4.11. Statistical Analysis

All statistical analyses and dose–response curve fittings were conducted using OriginPro 2022 and GraphPad Prism version 10. Differences between treatment groups were considered statistically significant at *p* ≤ 0.05. Redundancy analysis (RDA) and Pearson correlation analyses were performed using Canoco^©^ 5.0. Descriptive results are presented as mean ± standard deviation (SD).

## Figures and Tables

**Table 1:** Toxicity responses of *Artemia salina* (LC_50_), *Daphnia similis* (EC(I)_50_), and *Parhyale hawaiensis* (LC_50_) species to different tested extracts from *Microcystis aeruginosa* CCIBt3106 (a non-microcystin-producing strain).

Extracts	*Artemia salina*	*Daphnia similis*	*Parhyale hawaiensis*
	EC_50_/LC_50_ (95% CI) (µg mL^−1^)		
Aqueous extract	>1000	740 (490–790)	800 (770–850)
MeOH 50% extract	>1000	940 (540–1380)	660 (610–720)
MeOH 100% extract	>1000	>1000	600 (490–880)
DCM: MeOH 1:1 extract	>1000	>1000	>1000

EC(I)_50_: effective concentration that immobilizes 50% of organisms; LC_50_: lethal concentration; CI = confidence interval; MeOH = methanol; DCM = dichloromethane.

**Table 2:** Putative compounds annotated in extracts and fractions of *M. aeruginosa* CCIBt3106 based on LC-MS/MS (HR-QTOF) analysis, with annotation levels assigned according to the confidence framework of Schymanski et al. [27].

Samples	Mean *m*/*z* [M + H]^+^ (Measured)	Mean RT (min)	Putative Formula	Putative Compound	*m*/*z* Error (ppm)	Annotation Level *	Reference
All extracts and fractions	269.0889	1.48	C_10_H_12_N_4_O_5_	Inosine	1.152037	2a	Srinivasan et al. [28].
MeOH 100%, MeOH 50%, DCM:MeOH 1:1, F1–F4 (H_2_O and MeOH)	414.1622	3.1	C_16_H_23_N_5_O_8_	Microcystbiopterin B or E	−0.700208	2b	Lifshits et al. [29].
All extracts and fractions	591.3587	5.02	C_31_H_50_N_4_O_5_S	New microginin?	1.149896	2b	Welker et al. [30].

* Annotation levels follow the system proposed by Schymanski et al. [27]: Level 2a = probable structure, library spectrum match; Level 2b = probable structure, diagnostic evidence without reference spectrum.

## Data Availability

The original contributions presented in this study are included in the article. Further inquiries can be directed to the corresponding authors.

## Supplementary Materials

The following supporting information can be downloaded at: https://www.mdpi.com/article/10.3390/toxins17070321/s1. Table S1: *Biomass production metrics for Microcystis aeruginosa CCIBt3106.* Summary of total dry biomass obtained over one year of cultivation, average biomass yield per liter, and standard deviation. The final cell density is shown after approximately 40 days of growth, along with the normalized cell count per milligram of dry biomass. Table S2: Extraction yields of *Microcystis aeruginosa* CCIBt3106 biomass using solvents of varying polarity. Mean extraction yields and standard deviations are shown for each solvent used: ultrapure water, 50% methanol, 100% methanol, and dichloromethane–methanol (1:1, *v*/*v*). Values reflect differences in the solubility profile of cyanobacterial metabolites. Table S3: Representative *m*/*z* values of LC-MS/MS features detected in *Microcystis aeruginosa* CCIBt3106 extracts and fractions, grouped by chemical class according to GNPS and ClassyFire annotations. For each class, the table lists example precursor *m*/*z* values, sample occurrence (fractions and control, only spring water), and classification based on the presence of toxic, non-toxic, or both types of fractions (as determined by *Daphnia similis* immobilization assays). Table S4: List of oligopeptides exclusively detected in toxic fractions of *M. aeruginosa* CCIBt3106 based on MS/MS spectra. Precursor ion *m*/*z* values are listed alongside retention time and key diagnostic fragments. Figure S1: Molecular network constructed from LC-MS/MS features of *M. aeruginosa* CCIBt3106 extracts obtained with solvents of increasing polarity. Nodes represent distinct molecular features, with edges reflecting MS^2^ similarity. Node colors represent extract origin: dark green for control (spring water), orange for H_2_O extract, cyan for the DCM: MeOH 1:1 extract, purple for the MeOH 50% extract, and light lavender for the MeOH 100% extract. Each node may contain a pie chart indicating detection across multiple extracts. The network illustrates the solvent-dependent distribution of metabolites and the overlap among chemical profiles. Figure S2: Distribution of chemical features across all aqueous and methanol fractions. Boxplots represent the total number of features detected via LC-MS/MS (HR-QTOF), illustrating variability in metabolite richness among fractions. Figure S3: MS^2^ spectral comparison for inosine (precursor *m*/*z* 269.0877). Top: reference spectrum from the GNPS library (accession CCMSLIB00005720607) annotated with major fragment ions. Bottom: experimental MS^2^ spectrum from *Microcystis aeruginosa* CCIBt3106 extract. Red dashed lines highlight coincident fragment ions detected in both spectra: *m*/*z* 82.04, 94.04, 110.04, 119.04, 137.05, supporting annotation confidence Level 2a according to Schymanski et al. [27]. Figure S4: Relative abundance of the features *m*/*z* 269.088 (blue), 414.162 (orange), and 591.359 (green), and their association with toxicity responses in aquatic bioindicators. The bar plot represents the mean relative intensity of the ions across four different extracts obtained from *Microcystis aeruginosa* CCIBt3106 (H_2_O, MeOH 50%, MeOH 100%, and DCM: MeOH). Overlaid line plots represent the relative toxicity (expressed as 1/EC_50_ or 1/LC_50_) for *Daphnia similis*, *Parhyale hawaiensis*, and *Artemia salina*, highlighting the inverse relationship between EC_50_ and LC_50_ values and toxicity. Figure S5: Comparative abundance of the feature *m*/*z* 269.088 in fractionated samples from aqueous and MeOH 50% extracts and its association with toxicity responses in aquatic bioindicators. Bar plots show relative intensity (×10^4^) in each fraction (F1-F6) and full extract, while lines indicate the percent immobilization of *D. similis* after 24 h of exposure to each sample. Figure S6: MS^2^ spectral comparison for a putative microcystbiopterin (precursor *m*/*z* 414.1623). Top: reference MS^2^ spectrum for biopterin from the MoNA database (accession MoNA010961), annotated with major fragment ions. Bottom: experimental MS^2^ spectrum from *Microcystis aeruginosa* CCIBt3106 extract. Red dashed lines indicate fragment ions coincident in both spectra, *m*/*z* 124.05, 147.07, 178.07, 192.09, 220.08, and 238.09, supporting structural similarity. Figure S7: Comparative abundance of the feature *m*/*z* 414.16 in fractionated samples from aqueous and MeOH 50% extracts and its association with toxicity responses in aquatic bioindicators. Bar plots show relative intensity (×10^4^) in each fraction (F1–F6) and full extract, while lines indicate the percent immobilization of *D. similis* after 24 h of exposure to each sample. Figure S8: MS^2^ spectrum of the feature with *m*/*z* 591.3602 [M + H]^+^ (RT 5.1 min), annotated as a putative new microginin. Diagnostic fragment ions include *m*/*z* 128.9621 and 345.9130, consistent with known microginin structures [30]. Spectrum acquired from the MeOH 100% extract. Figure S9: Comparative abundance of the feature *m*/*z* 591.359 in fractionated samples from aqueous and MeOH 50% extracts and its association with toxicity responses in aquatic bioindicators. Bar plots show relative intensity (×10^4^) in each fraction (F1–F6) and full extract, while lines indicate the percent immobilization of *D. similis* after 24 h of exposure to each sample. Figure S10: MS/MS spectra and molecular network nodes of putatively annotated oligopeptides detected exclusively in toxic fractions of *Microcystis aeruginosa* CCIBt3106 extracts. Each panel shows the fragmentation spectrum corresponding to a feature (*m*/*z* labeled in red) with characteristic immonium and fragment ions consistent with amino acid substructures. The network visualization (left) highlights the connectivity of structurally related peptides. Figure S11: Additional MS/MS spectra of oligopeptide features exclusive to toxic fractions of *Microcystis aeruginosa* CCIBt3106. Fragmentation patterns include diagnostic ions indicative of modified residues (e.g., Ahp and Me-Tyr), reinforcing the presence of bioactive cyanopeptides with potential ecological relevance. The network diagram (left) illustrates cluster relationships among these features based on spectral similarity.

## Abbreviations

The following abbreviations are used in this manuscript:

ASW	Artificial seawater
BOD	Biological oxygen demand
CCIBt	Algae, Cyanobacteria, and Fungi Culture Collection of the Botany Institute
CI	Confidence interval
DMSO	Dimethyl sulfoxide
EC_50_/LC_50_	Median effective/lethal concentration (50%)
EPA	Environmental Protection Agency
F1–F6	Fraction numbers (0–100% IPA gradient)
GNPS	Global Natural Products Social Molecular Networking
HR-QTOF-MS/MS	High-resolution quadrupole time-of-flight tandem mass spectrometry
IPA	Isopropyl alcohol
LC-MS/MS	Liquid chromatography coupled with tandem mass spectrometry
MeOH	Methanol
MCs	Microcystins
NMR	Nuclear magnetic resonance
RDA	Redundancy analysis
RT	Retention time
SD	Standard deviation
TDI	Tolerable daily intake
WHO	World Health Organization
