# Supplementary material for: Unraveling the Toxicity of a Non-Microcystin-Producing Strain (CCIBt3106) of Microcystis aeruginosa: Ecotoxicological Effects on Aquatic Invertebrates
Source: Toxins (Basel). 2025 Jun 24;17(7):321. doi: 10.3390/toxins17070321 (PMC12299722; doi:10.3390/toxins17070321)
Supplement: Supplementary file 1 [file toxins-17-00321-s001.zip › toxins-3650062-supplementary.pdf]

toxins

MDPI

Supplementary Material

Table S1. Biomass production metrics for *Microcystis aeruginosa* CCIBt3106. Summary of total dry biomass obtained over one year of cultivation, average biomass yield per liter, and standard deviation. The final cell density is shown after approximately 40 days of growth, along with the normalized cell count per milligram of dry biomass.

Metric	Value
Total Biomass (g)	43.31
Average Biomass Yield (mg/L)	122.69
Standard Deviation (mg/L)	47.22
Final Cell Density (cells/mL)	4.22×10^7
Normalized Cell Count (cells/mg dry biomass)	1.00×10^8

Table S2. Extraction yields of *Microcystis aeruginosa* CCIBt3106 biomass using solvents of varying polarity. Mean extraction yields and standard deviations are shown for each solvent used: ultrapure water, 50% methanol, 100% methanol, and dichloromethane: methanol (1:1, v/v). Values reflect differences in the solubility profile of cyanobacterial metabolites.

**Extraction Solvent**	**Mean Yield (%)**	**Standard Deviation (%)**
Ultrapure Water	67.51	31.01
Methanol 50%	31.15	19.81
Methanol 100%	15.89	3.71
Dichloromethane: Methanol (1:1, v/v)	11.94	7.77

 https://doi.org/10.3390/toxins17070321

Table S3. Representative *m/z* values of LC-MS/MS features detected in *Microcystis aeruginosa* CCIBt3106 extracts and fractions, grouped by chemical class according to GNPS and ClassyFire annotations. For each class, the table lists example precursor *m/z* values, sample occurrence (fractions and control, only spring water), and classification based on presence in toxic, non-toxic, or both types of fractions (as determined by *Daphnia similis* immobilization assays).

Chemical Class	m/z	Samples	Fractions Toxicity
Benzene and substituted derivatives	393.205; 404.341; 485.341	F3 and F5-F6 H_2O; F5-F6 MeOH 50%	Toxic
Carboxylic acids and derivatives	438.198; 527.244; 579.271; 650.308; 780.304	F3-F4 H_2O and F3-F4 MeOH 50%	Toxic
Fatty Acyls	436.261; 450.24; 556.49	F3-F6 H_2O and F3-F5 MeOH 50%	Toxic
Flavonoids	373.255	F4-F6 H_2O and F4-F5 MeOH 50%	Toxic
Organooxygen compounds	278.885; 479.182; 564.353	F2 and F4-F5 H_2O; F2-F5 MeOH 50%	Toxic
Piperidines	419.211	F4 H_2O and F4 MeOH 50%	Toxic
Prenol lipids	253.214; 432.234	F4 H_2O; F4 and F6 MeOH 50%	Toxic
Steroids and steroid derivatives	**269.208**; 271.224	F4 H_2O and F4 MeOH 50%	Toxic
Tetrapyrroles and derivatives	887.558	Pool	Toxic
Benzene and substituted derivatives	391.281	F1 H_2O; F1 MeOH 50%, and CTL	Non-toxic
Carboxylic acids and derivatives	307.081; 371.601; 613.153	F1 MeOH 50%	Non-toxic

**Chemical Class**	**m/z**	**Samples**	**Fractions Toxicity**
Coumarins and derivatives	568.473	F1 MeOH 50% and CTL	Non-toxic
Organooxygen compounds	425.172; 491.236; 497.193	F1-F2 H_2O and F1 MeOH 50%	Non-toxic
Steroids and steroid derivatives	664.449	CTL	Non-toxic
Benzene and substituted derivatives	333.101; 365.132; 393.293; 394.289; 425.283; 427.297; 429.229; 600.462	F1-F6 H_2O; F1-F6 MeOH 50%, and CTL	Both
Carboximidic acids and derivatives	482.488	F1-F6 H_2O; F1-F6 MeOH 50%, and CTL	Both
Carboxylic acids and derivatives	307.081; 311.12; 618.254	F2-F3 H_2O; F1-F4 MeOH 50%	Both
Coumarins and derivatives	554.545; 569.526; 570.539; 579.529; 584.519; 598.534; 612.55	F1-F6 H_2O; F1-F6 MeOH 50%, and CTL	Both
Fatty Acyls	344.313; 360.209; 361.217; 372.344; 402.317; 434.245; 466.493; 526.514; 542.508; 543.509; 570.503; 597.557; 598.57	F1-F6 H_2O; F1-F6 MeOH 50%, and CTL	Both
Flavonoids	212.018; 265.088; 280.865; 924.26	F1-F6 H_2O; F1-F6 MeOH 50%, and CTL	Both
Glycerolipids	584.555	F1, F3, F6 MeOH 50%, and CTL	Both

**Chemical Class**	**m/z**	**Samples**	**Fractions Toxicity**
Imidazopyrimidines	268.101; 284.096	F1-F6 H_2O; F1-F6 MeOH 50%	Both
Linear 1,3-diarylpropanoids	313.271	F1-F6 H_2O; F1-F6 MeOH 50%, and CTL	Both
Organooxygen compounds	294.115; 424.177; 526.754; 688.513; 732.539; 776.565	F1-F6 H_2O; F1-F6 MeOH 50%, and CTL	Both
Phenol ethers	502.369	F1-F6 H_2O; F1-F6 MeOH 50%, and CTL	Both
Prenol lipids	276.105; 437.189	F1-F6 H_2O; F1-F6 MeOH 50%, and CTL	Both
Pteridines and derivatives	**414.158**	F1-F4 and F6 H_2O; F1-F4 and F6 MeOH 50%	Both
Steroids and steroid derivatives	387.189; 663.446; 665.425; 701.423	F1-F6 H_2O; F1-F6 MeOH 50%, and CTL	Both

Table S4. List of oligopeptides exclusively detected in toxic fractions of *M. aeruginosa* CCIBt3106 based on MS/MS spectra. Precursor ion *m/z* values are listed alongside retention time and key diagnostic fragments.

**Retention Time (min)**	**Precursor (*m/z*)**	**Fragments (*m/z*) and %**
**3.9**	573.318	84 (1.5%) Lys; 86.0968 (2.3%) Leu/Ile; 102.0553 (5.6%) Glu, 120.0809 (100.0%) Phe
**4.3**	527.244	84 (1.5%) Lys; 86.097 (8.9%) Leu/Ile; 120.081(85.0%) Phe; 129.082 (4.7%) Glu or derivatives; 136.076 (100.0%) Tyr
**3.7**	502.282	70.067 (1.5%) Pro; 84.045 (6.8%) Lys, 86.097 (24.7%) Leu/Ile
**4.6**	571.3	84.084 (1.7%) Lys; 86.096 (3.3%) Leu/Ile; 120.08 (6.7%) Phe; 166 (100%) Phe
**4.5**	574.321	84.084 (1.7%) Lys; 86.096 (3.3%) Leu/Ile; 120.08 (6.7%) Phe; 166 (100%) Phe
**3.9**	461.239	86.098 (4.1%) Leu/Ile; 166 (100%) Phe
**2.6**	490.246	84.052 (4.4%) Lys; 102.055 (9.2%) Glu; 120.081 (11.3%) Phe; 140.071 (36.5%) Tyr or related
**2.3**	419.211	84.052 (13.3%) Lys; 140.071 (30.7%) Tyr or related
**4.9**	608.379	84.083 (15.3%) Lys; 86.097 (17.6%) Leu/Ile; 120.081 (57.9%) Phe
**5.1**	696.43	84.083 (15.3%) Lys; 86.097 (17.6%) Leu/Ile; 89.060 (100.0%) Ala/Gly-related; 120.081 (57.9%) Phe; 129.04 (14.3%) Lys; 133.08 (86.6%) Thr/Ahp
**3.2**	534.287	86.097 (21.9%) Leu/Ile; 120.081 (100.0%) Phe
**4.3**	605.323	70.067 (1.3%) Pro; 86.097 (4.5%) Leu/Ile; 120.081 (49.3%) Phe; 136.076 (5.1%) Tyr
**5.5**	693.389	84.046 (1.4%) Lys ; 86.097 (8.2%) Leu/Ile; 120.081 (77.4%) Phe
**4.3**	433.241	70.07 (1.5%) Pro; 84.046 (12.4%) Lys; 86.098 (18.2%) Leu/Ile; 120.08 (59.1%) Phe; 166 (6.1%) Phe; 169.119 (100.0%) 197.128 (17.6%) typical of cyclic peptides
**4.3**	650.308	84.047 (1.5%) Lys; 86.099 (2.3%) Leu/Ile; 102.054 (5.6%) Glu; 120.081 (100.0%) Phe
**4.1**	579.271	84.046 (2.6%); 86.096 (5.1%) Leu/Ile; 102.056 (7.7%) Glu; 120.081 (100.0%) Phe

Lys: Lysine; Leu/Ile: Leucine / Isoleucine; Glu: Glutamic acid; Phe: Phenylalanine; Tyr: Tyrosine; Pro: Proline; Ala/Gly: Alanine / Glycine (fragment); Thr/Ahp: Threonine + Ahp (modified).

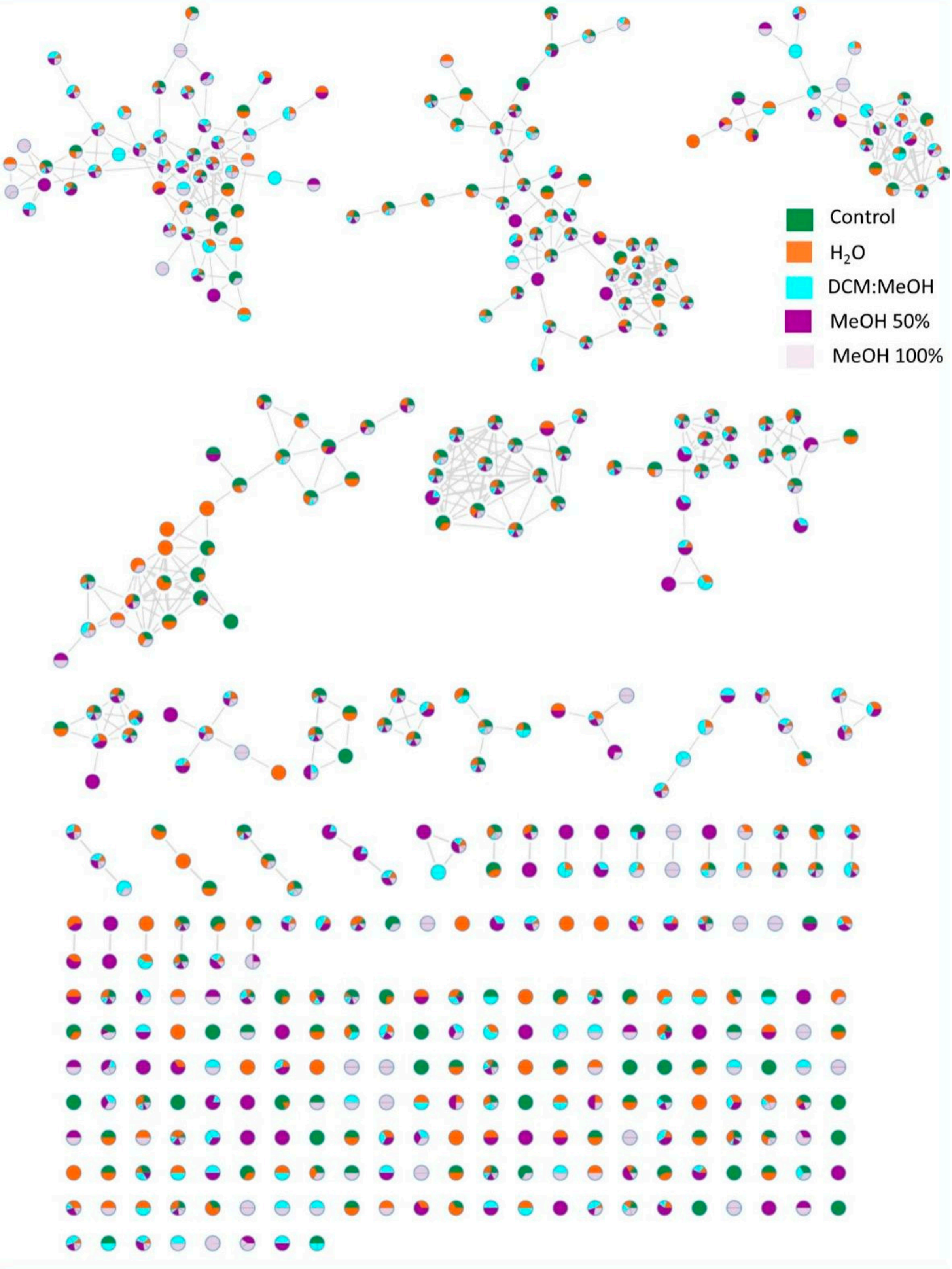

Figure S1. Molecular network constructed from LC-MS/MS features of *M. aeruginosa* CCIBt3106 extracts obtained with solvents of increasing polarity. Nodes represent distinct molecular features, with edges reflecting MS^2 similarity. Node colors represent extract origin: dark green for control (spring water), orange for H_2O extract, cyan for DCM: MeOH 1:1 extract, purple for MeOH 50% extract, and light lavender for MeOH 100% extract. Each node may contain a pie chart indicating detection across multiple extracts. The network illustrates the solvent-dependent distribution of metabolites and the overlap among chemical profiles.

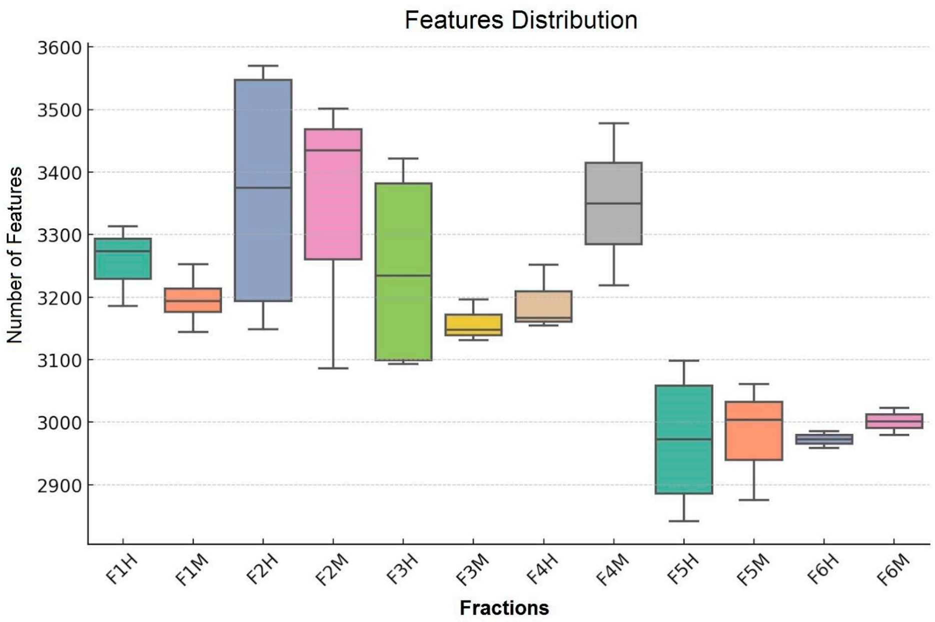

Figure S2. Distribution of chemical features across all aqueous and methanol fractions. Boxplots represent the total number of features detected via LC-MS/MS (HR-QTOF), illustrating variability in metabolite richness among fractions.

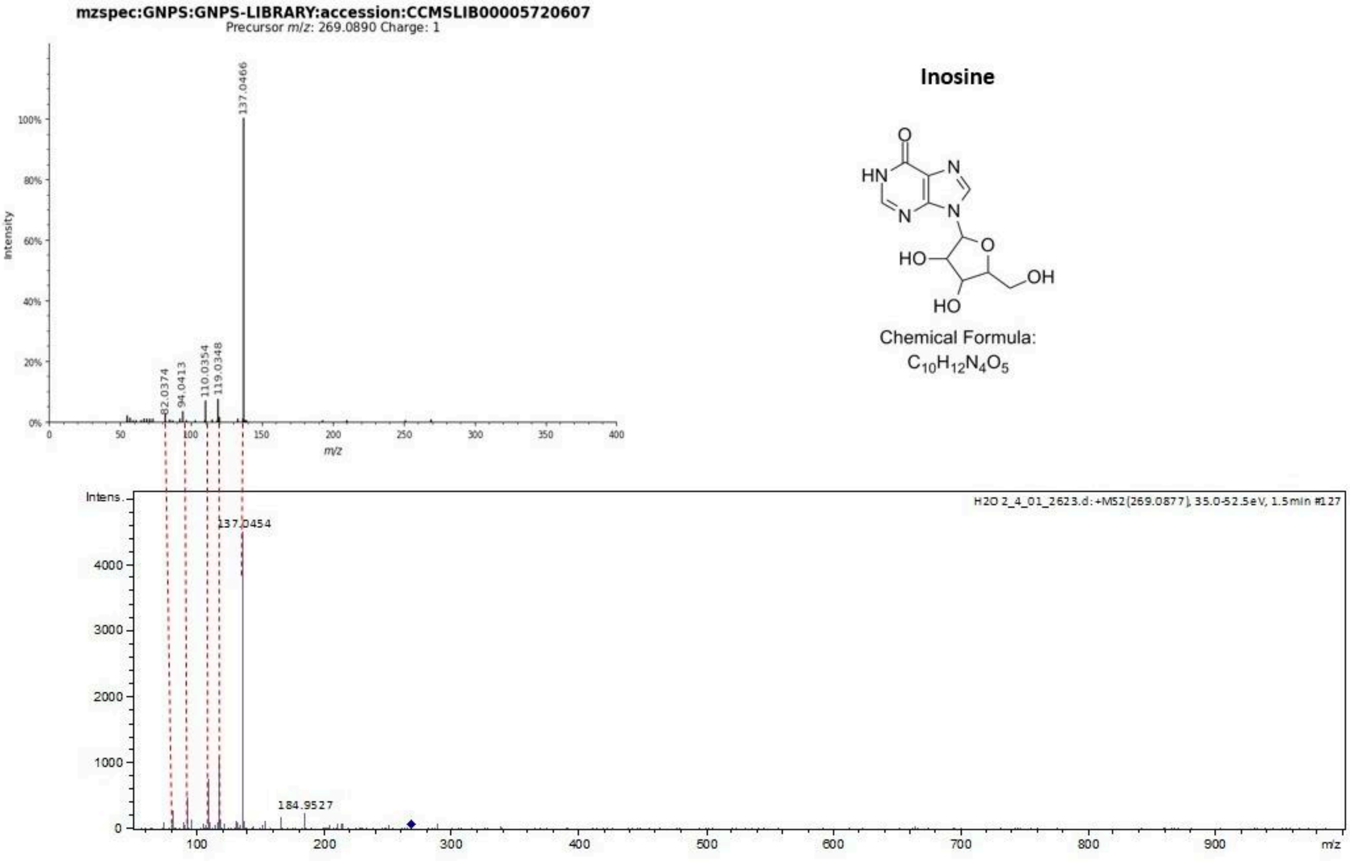

Figure S3. MS² spectral comparison for inosine (precursor *m/z* 269.0877). Top: reference spectrum from the GNPS library (accession CCMSLIB00005720607) annotated with major fragment ions. Bottom: experimental MS² spectrum from *Microcystis aeruginosa* CCIBt3106 extract. Red dashed lines highlight coincident fragment ions detected in both spectra: *m/z* 82.04, 94.04, 110.04, 119.04, 137.05, supporting annotation confidence level 2a according to Schymanski et al. (2014).

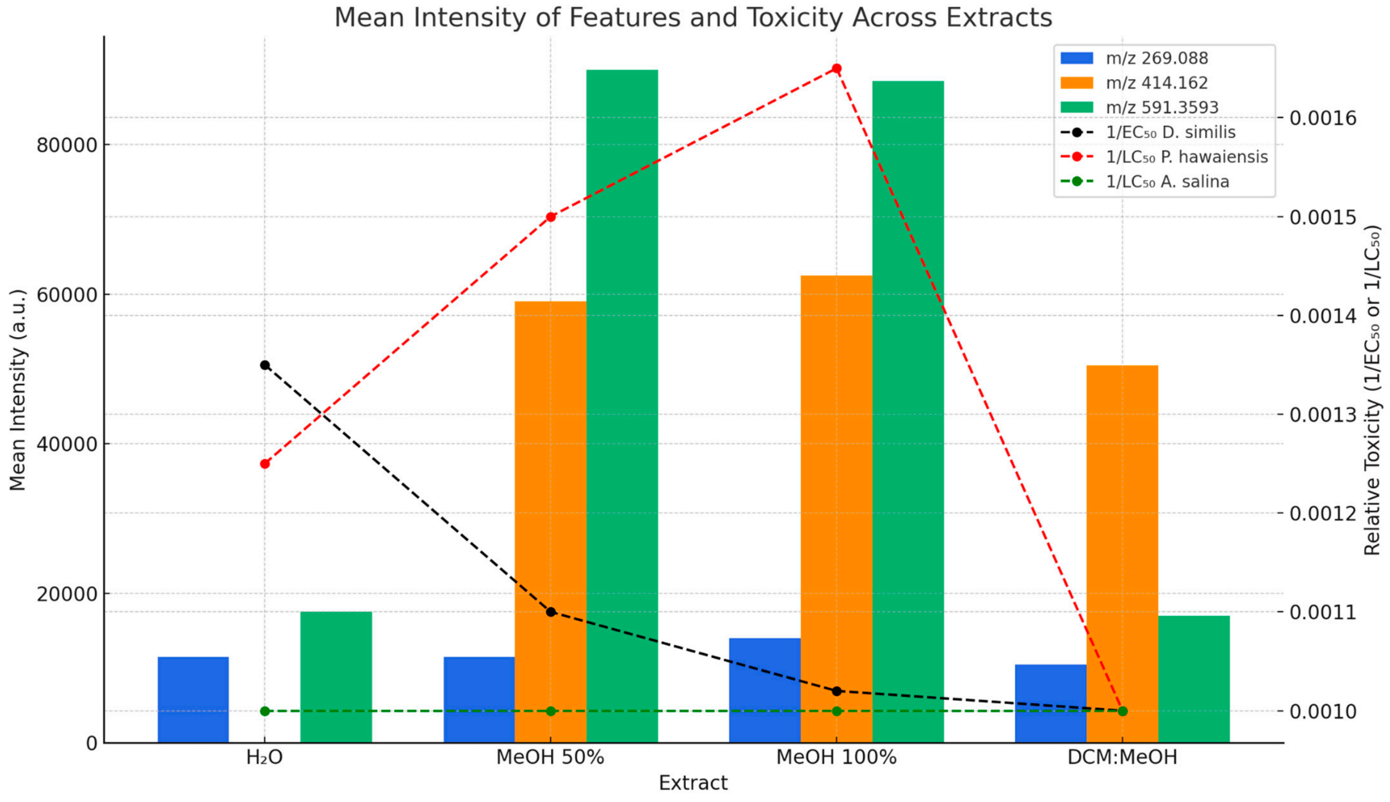

Figure S4. Relative abundance of the features *m*/*z* 269.088 (blue), 414.162 (orange), and 591.359 (green), and their association with toxicity responses in aquatic bioindicators. The bar plot represents the mean relative intensity of the ions across four different extracts obtained from *Microcystis aeruginosa* CCIBt3106 (H_2O, MeOH 50%, MeOH 100%, DCM: MeOH). Overlaid line plots represent the relative toxicity (expressed as $1/EC_{50}$ or $1/LC_{50}$) for *Daphnia similis*, *Parhyale hawaiensis*, and *Artemia salina*, highlighting the inverse relationship between EC_{50} and LC_{50} values and toxicity.

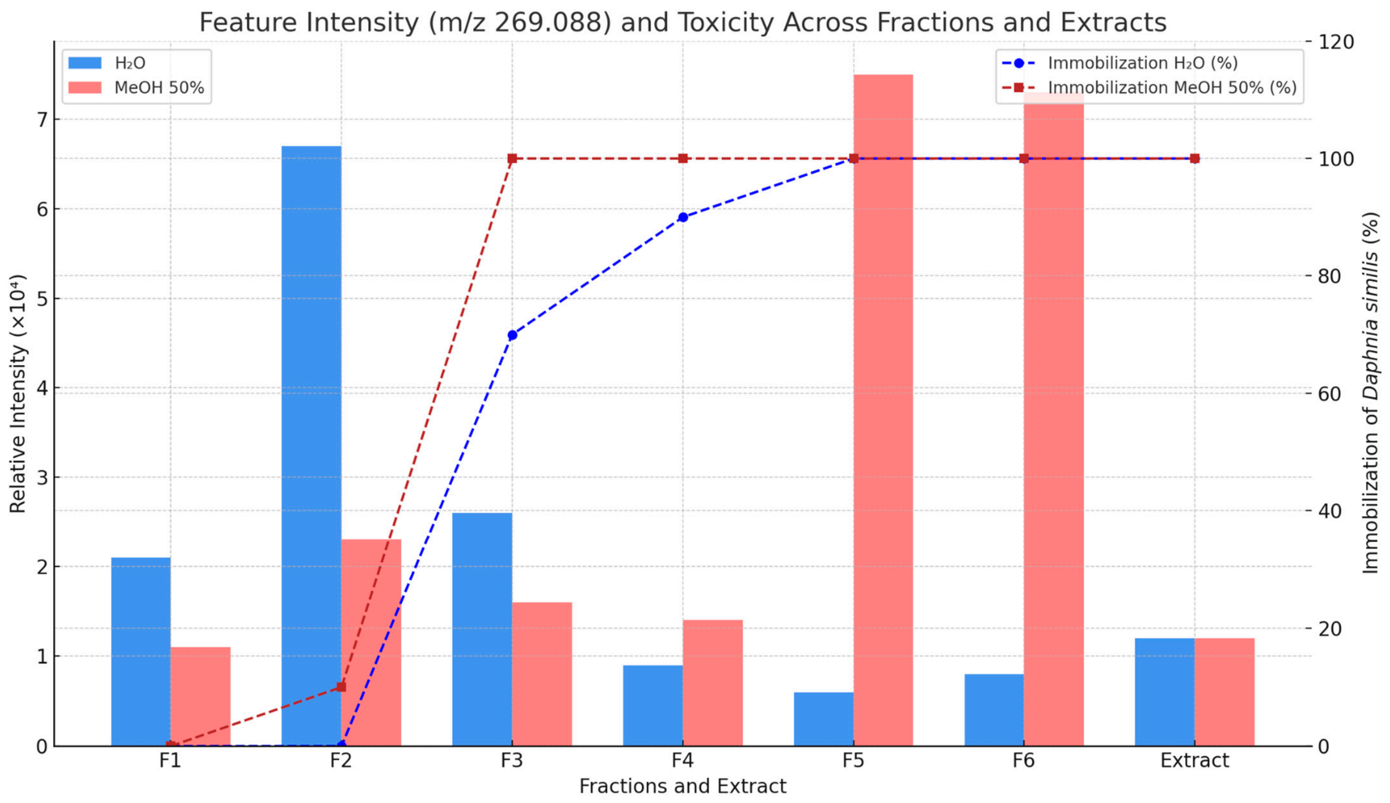

Figure S5. Comparative abundance of the feature *m*/*z* 269.088 in fractionated samples from aqueous and MeOH 50% extracts and its association with toxicity responses in aquatic bioindicators. Bar plots show relative intensity (×10^4) in each fraction (F1–F6) and full extract, while lines indicate the percent immobilization of *D. similis* after 24 hours of exposure to each sample.

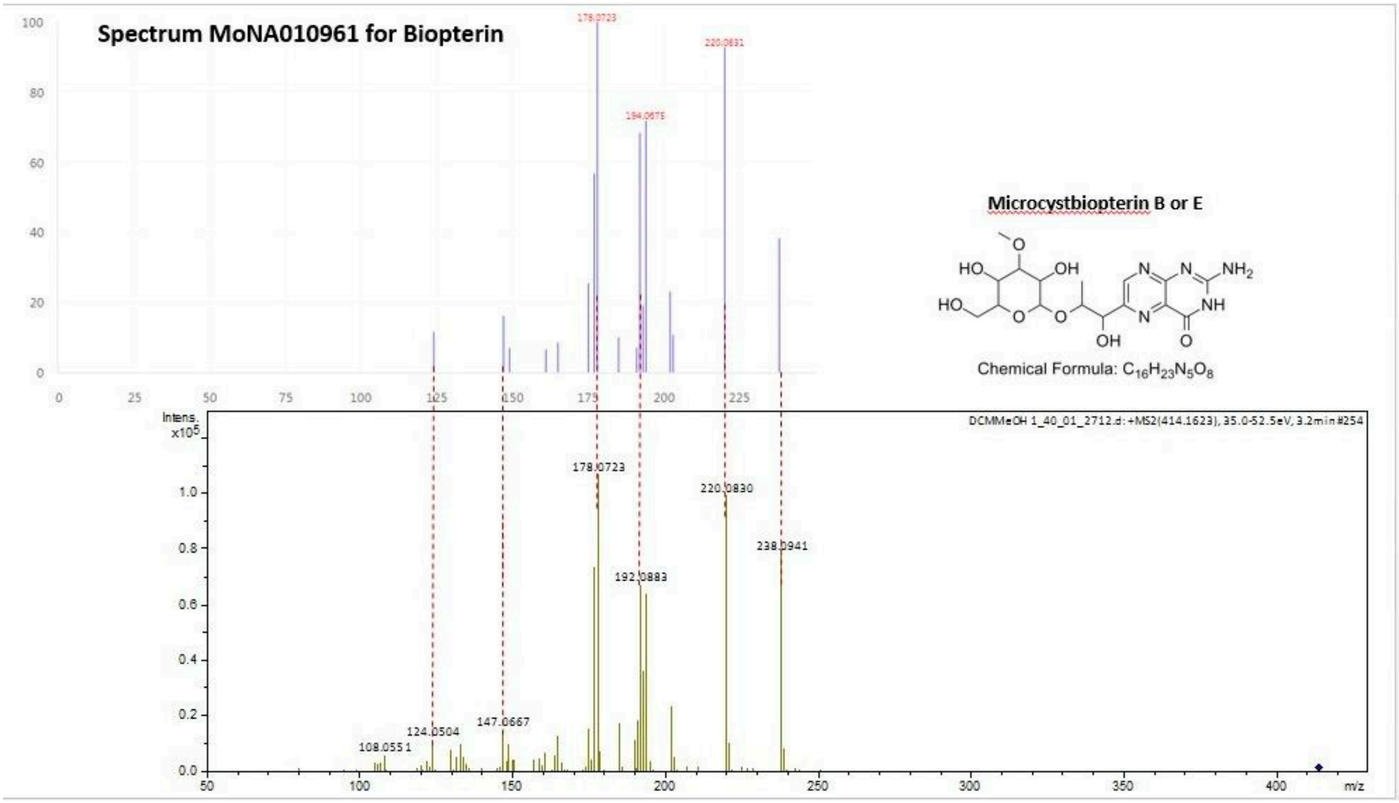

Figure S6. MS^2 spectral comparison for a putative microcystbiopterin (precursor *m/z* 414.1623). Top: reference MS^2 spectrum for biopterin from the MoNA database (accession MoNA010961), annotated with major fragment ions. Bottom: experimental MS^2 spectrum from *Microcystis aeruginosa* CCIBt3106 extract. Red dashed lines indicate fragment ions coincident in both spectra: *m/z* 124.05, 147.07, 178.07, 192.09, 220.08, and 238.09, supporting structural similarity.

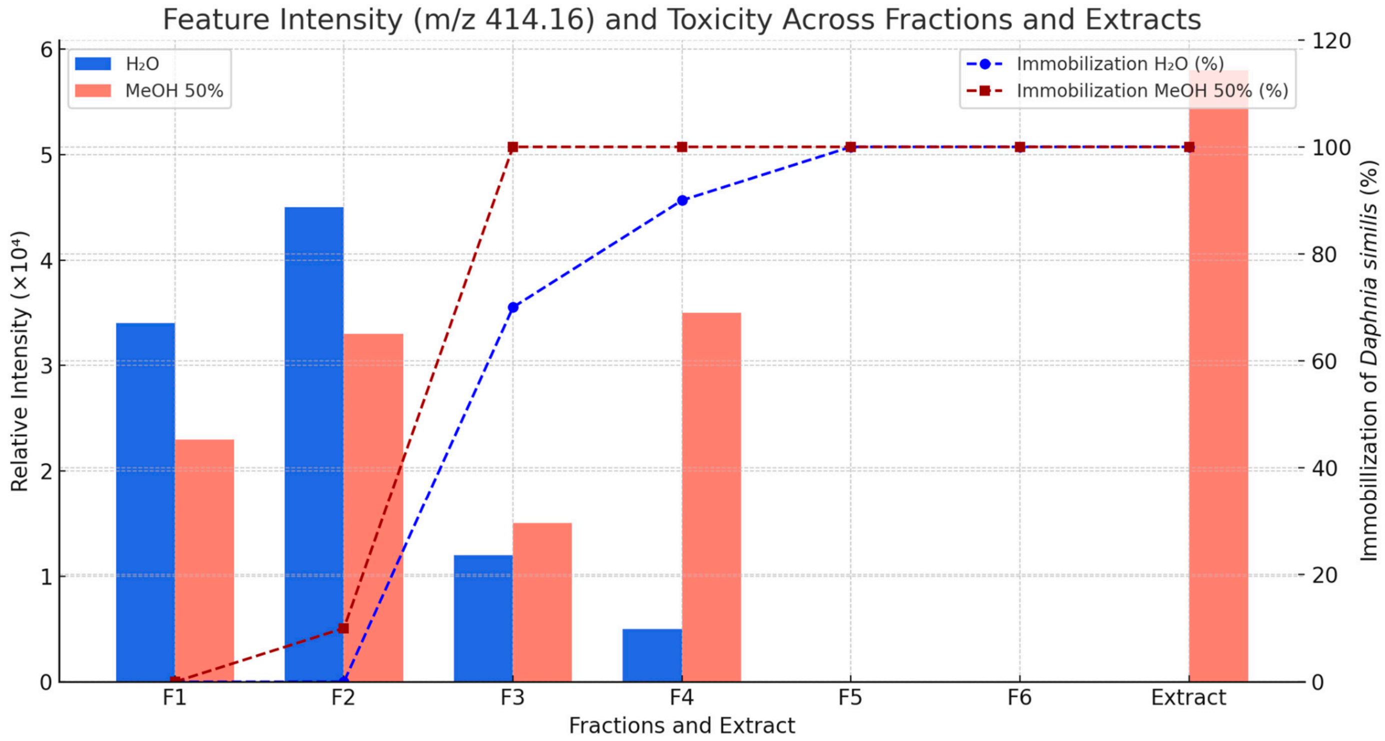

Figure S7. Comparative abundance of the feature *m*/*z* 414.16 in fractionated samples from aqueous and MeOH 50% extracts and its association with toxicity responses in aquatic bioindicators. Bar plots show relative intensity (×10^4) in each fraction (F1–F6) and full extract, while lines indicate the percent immobilization of *D. similis* after 24 hours of exposure to each sample.

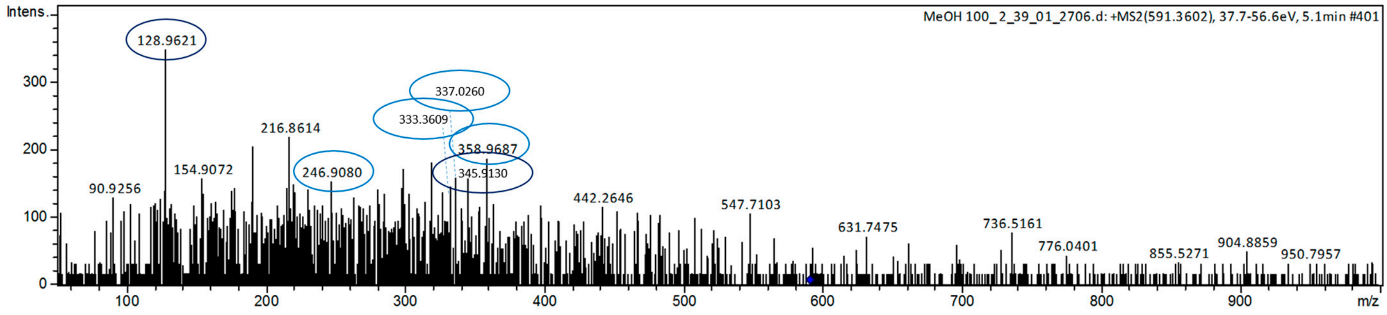

Figure S8. MS² spectrum of the feature with *m*/*z* 591.3602 [M+H]+ (RT 5.1 min), annotated as a putative new microginin. Diagnostic fragment ions include *m*/*z* 128.9621 and 345.9130, consistent with known microginin structures (Welker et al., 2006). Spectrum acquired from the MeOH 100% extract.

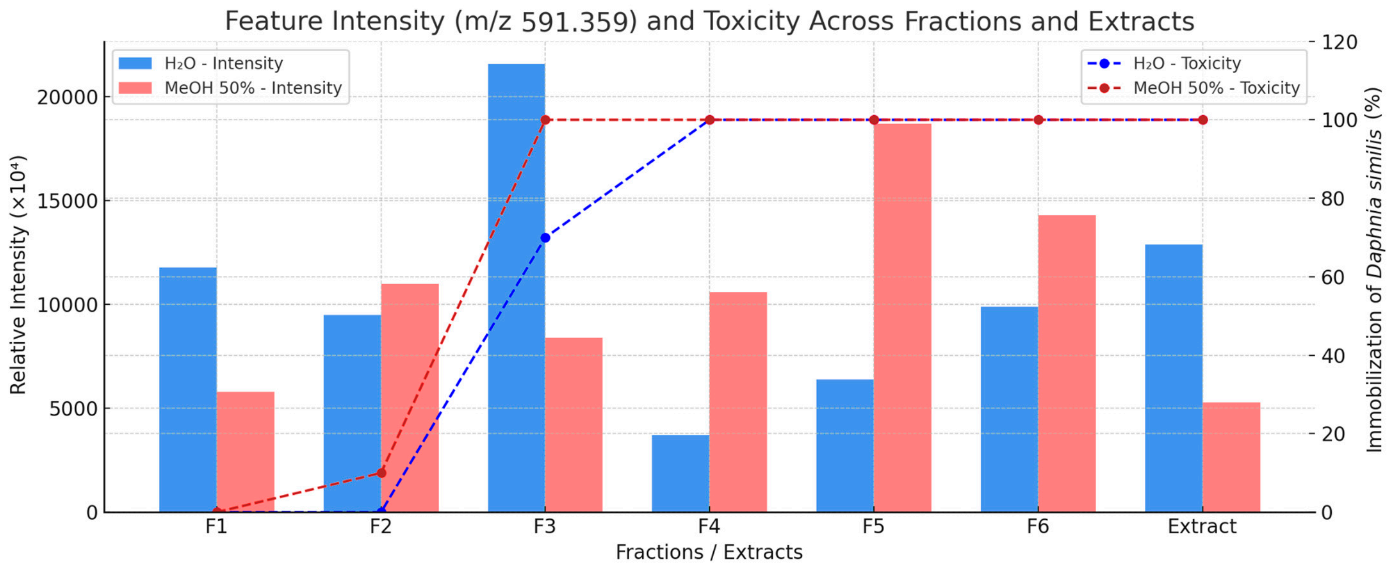

Figure S9. Comparative abundance of the feature *m*/*z* 591.359 in fractionated samples from aqueous and MeOH 50% extracts and its association with toxicity responses in aquatic bioindicators. Bar plots show relative intensity (×10^4) in each fraction (F1-F6) and full extract, while lines indicate the percent immobilization of *D. similis* after 24 hours of exposure to each sample.

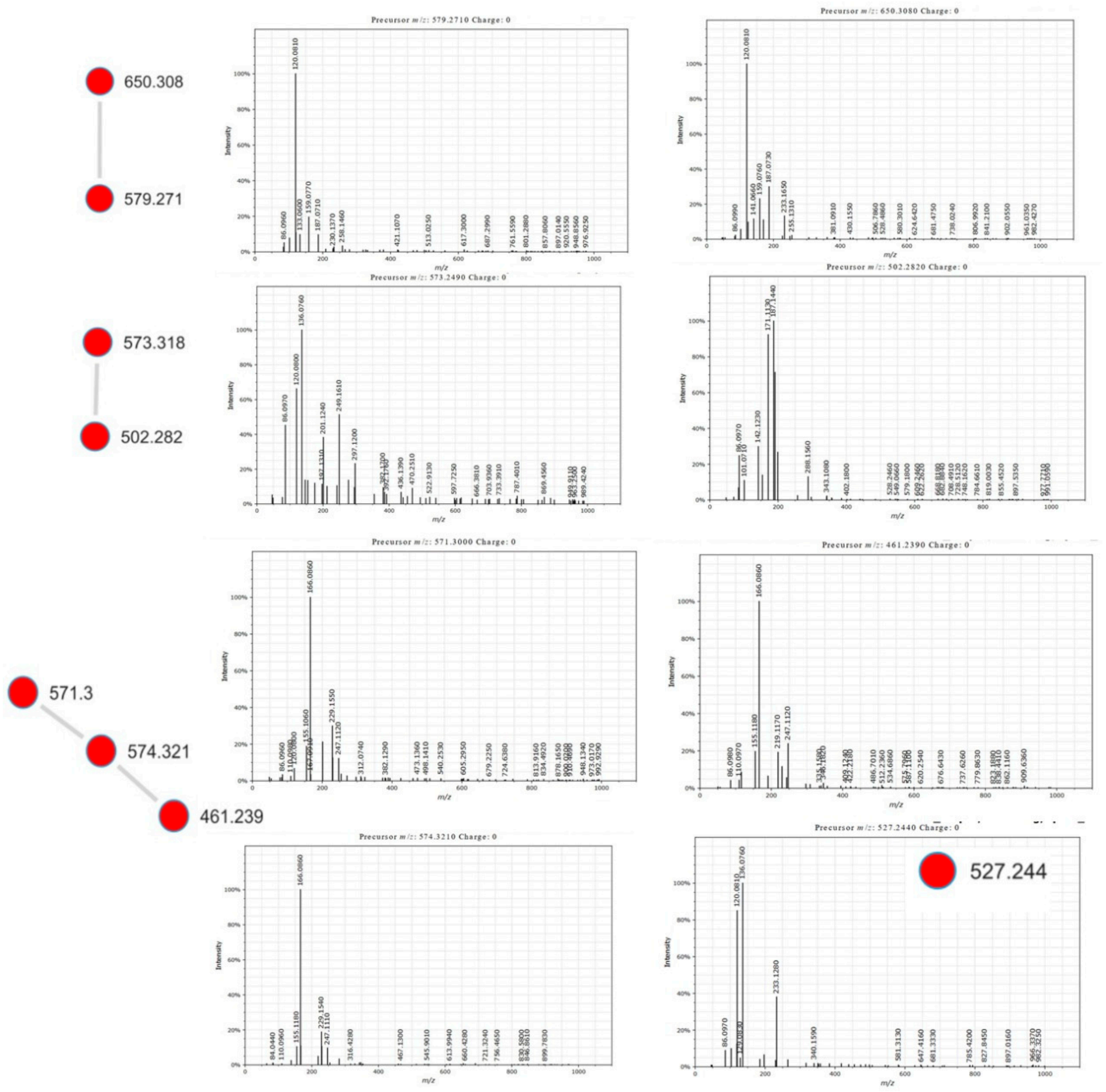

Figure S10. MS/MS spectra and molecular network nodes of putatively annotated oligopeptides detected exclusively in toxic fractions of *Microcystis aeruginosa* CCIBt3106 extracts. Each panel shows the fragmentation spectrum corresponding to a feature (*m/z* labeled in red) with characteristic immonium and fragment ions consistent with amino acid substructures. The network visualization (left) highlights the connectivity of structurally related peptides.

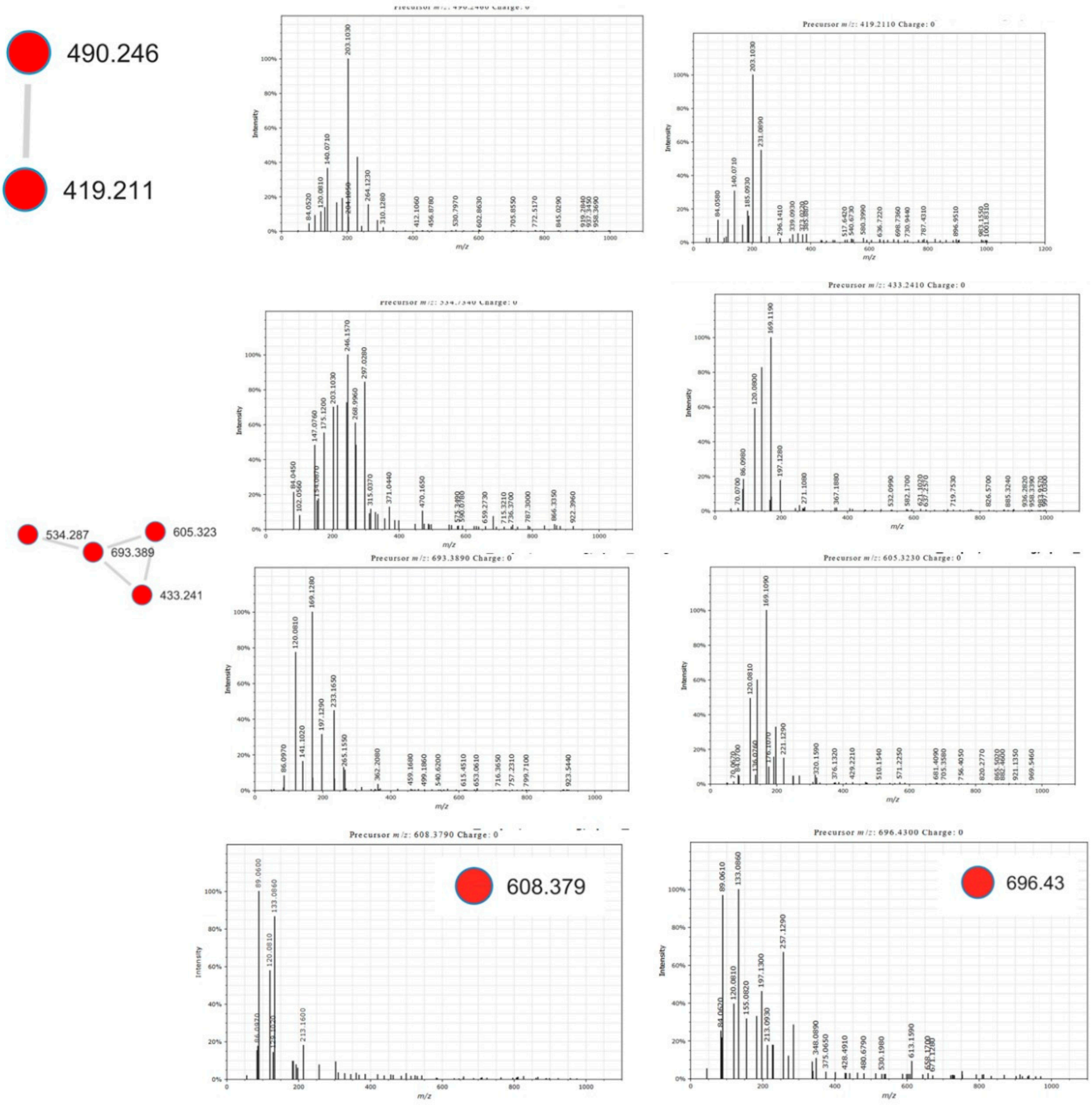

Figure S11. Additional MS/MS spectra of oligopeptide features exclusive to toxic fractions of *Microcystis aeruginosa* CCIBt3106. Fragmentation patterns include diagnostic ions indicative of modified residues (e.g., Ahp, Me-Tyr), reinforcing the presence of bioactive cyanopeptides with potential ecological relevance. The network diagram (left) illustrates cluster relationships among these features based on spectral similarity.
